# In vivo characterization of the critical interaction between the RNA exosome and the essential RNA helicase Mtr4 in *Saccharomyces cerevisiae*

**DOI:** 10.1093/g3journal/jkad049

**Published:** 2023-03-02

**Authors:** Maria C Sterrett, Daniela Farchi, Sarah E Strassler, Lawrence H Boise, Milo B Fasken, Anita H Corbett

**Affiliations:** Department of Biology, Emory University, Atlanta, GA 30322, USA; Biochemistry, Cell, and Developmental Biology Graduate Program, Emory University, Atlanta, GA 30322, USA; Department of Biology, Emory University, Atlanta, GA 30322, USA; Biochemistry, Cell, and Developmental Biology Graduate Program, Emory University, Atlanta, GA 30322, USA; Department of Biochemistry, Emory University, Atlanta, GA, 30322, USA; Department of Hematology and Medical Oncology, School of Medicine, Emory University, Atlanta, GA 30322, USA; Winship Cancer Institute, Emory University, Atlanta, GA 30322, USA; Department of Biology, Emory University, Atlanta, GA 30322, USA; Department of Biology, Emory University, Atlanta, GA 30322, USA

**Keywords:** RNA exosome, Mtr4, EXOSC2, Rrp4, RNA processing, multiple myeloma, RNA helicase

## Abstract

The RNA exosome is a conserved molecular machine that processes/degrades numerous coding and non-coding RNAs. The 10-subunit complex is composed of three S1/KH cap subunits (human EXOSC2/3/1; yeast Rrp4/40/Csl4), a lower ring of six PH-like subunits (human EXOSC4/7/8/9/5/6; yeast Rrp41/42/43/45/46/Mtr3), and a singular 3′-5′ exo/endonuclease DIS3/Rrp44. Recently, several disease-linked missense mutations have been identified in structural cap and core RNA exosome genes. In this study, we characterize a rare multiple myeloma patient missense mutation that was identified in the cap subunit gene *EXOSC2*. This missense mutation results in a single amino acid substitution, p.Met40Thr, in a highly conserved domain of EXOSC2. Structural studies suggest that this Met40 residue makes direct contact with the essential RNA helicase, MTR4, and may help stabilize the critical interaction between the RNA exosome complex and this cofactor. To assess this interaction in vivo, we utilized the *Saccharomyces cerevisiae* system and modeled the *EXOSC2* patient mutation into the orthologous yeast gene *RRP4*, generating the variant *rrp4-M68T.* The *rrp4-M68T* cells show accumulation of certain RNA exosome target RNAs and show sensitivity to drugs that impact RNA processing. We also identified robust negative genetic interactions between *rrp4-M68T* and specific *mtr4* mutants. A complementary biochemical approach revealed that Rrp4 M68T shows decreased interaction with Mtr4, consistent with these genetic results. This study suggests that the *EXOSC2* mutation identified in a multiple myeloma patient impacts the function of the RNA exosome and provides functional insight into a critical interface between the RNA exosome and Mtr4.

## Teaser

This study uses *Saccharomyces cerevisiae* to model a missense mutation in the RNA exosome gene *EXOSC2*, which was identified in a patient with multiple myeloma. This analysis of Rrp4, the budding yeast EXCOSC2 ortholog, provides insight into the interaction of Rrp4 with a key RNA exosome cofactor, the RNA helicase Mtr4.

## Introduction

**Fig. 1.**
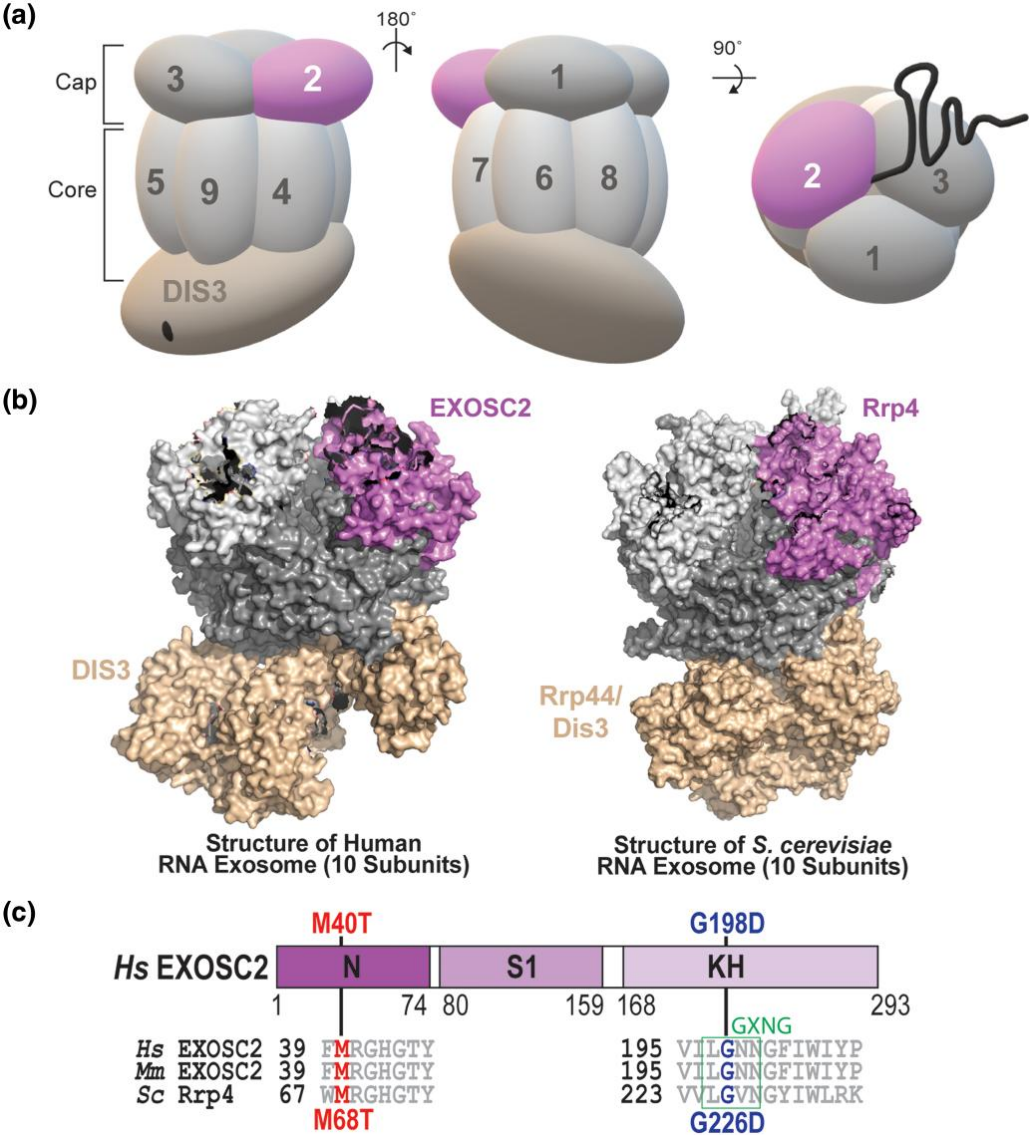
Overview of multiple myeloma-linked amino acid substitutions in the human cap subunit EXOSC2 of the RNA exosome. a) The RNA exosome is an evolutionary conserved ribonuclease complex composed of nine structural subunits (EXOSC1-9) and one catalytic subunit (DIS3) that form a “Cap” and “Core” ring-like structure. The three-subunit cap at the top of the complex is composed of EXOSC1/Csl4 (human/*S. cerevisiae*), EXOSC2/Rrp4, and EXOSC3/Rrp40 (labeled 1–3). The six-subunit core is composed of EXOSC4/Rrp41, EXOSC5/Rrp46, EXOSC6/Mtr3, EXOSC7/Rrp42, EXOSC8/Rrp43, and EXOSC9/Rrp45 (labeled 4–9). The DIS3/Dis3/Rrp44 catalytic subunit is located at the bottom of the complex. Together, the cap and core form a barrel-like structure through which RNA is threaded to the catalytic DIS3/Dis3/Rrp44 subunit. Recent missense mutation in the gene encoding the EXOSC2 cap subunit (pink, labeled "EXOSC2") has been identified in patients presenting with multiple myeloma. b) Structural models of the human nuclear RNA exosome (left) (PDB 6D6R) (Weick *et al*. 2018) and the *S. cerevisiae* nuclear RNA exosome (right) (PDB 6FSZ) (Schuller *et al*. 2018) are depicted with the cap subunit EXOSC2/Rrp4 labeled and colored. c) Domain structure of EXOSC2/Rrp4. This cap subunit is composed of three domains: an N-terminal domain, a putative RNA binding S1 domain, and a C-terminal putative RNA binding KH (K homology) domain. A conserved “GxNG” motif identified in the KH domain is boxed in green (Oddone *et al*. 2007). The position of the disease-linked amino acid substitutions in human EXOSC2 is depicted above the domain structures. The amino acid substitution (p.Met40Thr) that we report in a multiple myeloma patient is shown in red. An amino acid substitution (p.Gly198Asp) linked to SHRF is shown in blue (Di Donato *et al*. 2016). Sequence alignments of EXOSC2/Rrp4 orthologs from *Homo sapiens* (*Hs*), *Mus musculus* (*Mm*), and *S. cerevisiae* (*Sc*) below the domain structures show the highly conserved residues altered in disease in red and blue and the conserved sequences flanking these residues in gray.

The RNA exosome is a highly conserved exo/endonuclease complex that has an essential role in 3′ to 5′ processing and degradation of nearly every species of RNA (Schneider and Tollervey 2013; Zinder and Lima 2017). First identified in *Saccharomyces cerevisiae* in a screen for ribosomal RNA processing (*rrp*) mutants (Mitchell *et al*. 1996; Mitchell *et al*. 1997), the RNA exosome is essential in all organisms studied thus far (Mitchell *et al*. 1997; Lorentzen *et al*. 2007; Hou *et al*. 2012; Lim *et al*. 2013; Pefanis *et al*. 2014). In addition to ribosomal RNA precursors, the RNA exosome processes a variety of small non-coding RNAs (ncRNAs), including small nuclear RNAs (snRNAs) and small nucleolar RNAs (snoRNAs) (Allmang *et al*. 1999; Van Hoof *et al*. 2000; Kilchert *et al*. 2016; Fasken *et al*. 2020). The RNA exosome also plays roles in targeting RNA for degradation and decay, including non-functional or aberrant mRNAs and nuclear transcripts that result from pervasive transcription such as cryptic unstable transcripts (CUTs) in budding yeast or promoter upstream transcripts (PROMPTs) in humans (Wyers *et al*. 2005; Preker *et al*. 2008; Moore and Proudfoot 2009; Parker 2012; Schneider *et al*. 2012). The RNA exosome complex is composed of a 9-subunit structural core and a single exo/endonuclease [DIS3/DIS3L (human); Dis3/Rrp44 (budding yeast)]. As shown in Fig. 1a, the 9-subunit structural core is composed of three S1/KH cap subunits (EXOSC1/2/3; Csl4/Rrp4/Rrp40) and a lower ring of six PH-like subunits (EXOSC4/5/6/7/8/9; Rrp41/Rrp46/Mtr3/Rrp42/Rrp43/Rrp45). The nuclear RNA exosome has an additional 3′-5′ exonuclease, EXOSC10/Rrp6, that associates with the complex and aids in nuclear RNA targeting and processing (Briggs *et al*. 1998; Wasmuth *et al*. 2014). Structural studies demonstrate that the overall organization of the RNA exosome is conserved (Fig. 1b), suggesting not only evolutionary conservation of the RNA exosome function but structure as well (Makino *et al*. 2013; Schuller Jan *et al*. 2018; Weick *et al*. 2018). The vast array of targets and evolutionary conservation of the complex components indicates a fundamental role of the RNA exosome in several cellular processes, including but not limited to, maintaining genome integrity, translation, and cell differentiation through degradative and surveillance pathways (Ogami *et al*. 2018).

RNA exosome specificity for a broad set of target transcripts is conferred in part through interactions with cofactor proteins, which aid the RNA exosome in target recognition, RNA unwinding, degradation, and catalysis in both the nucleus and the cytoplasm (Zinder and Lima 2017; Fasken *et al*. 2020). Many nuclear RNA exosome cofactors were first characterized in budding yeast, including the Rrp6 obligate binding partner Rrp47, the cofactor Mpp6, and the essential 3′ to 5′ DExH box RNA helicase Mtr4 (De La Cruz *et al*. 1998; Mitchell *et al*. 2003; Lacava *et al*. 2005; Vaňáčová *et al*. 2005; Milligan *et al*. 2008), with orthologs now identified in the mammalian system (C1D, MPH6, and MTR4/MTREX) (Zinder and Lima 2017). Structural studies of the budding yeast and mammalian RNA exosome reveal that Rrp6/EXOSC10, Rrp47/C1D, and Mpp6/MPH6 interact with the complex through conserved interfaces that form composite sites for interactions with other cofactors such as Mtr4/MTR4/MTREX (Schuch *et al*. 2014; Falk *et al*. 2017; Wasmuth *et al*. 2017; Schuller *et al*. 2018; Weick *et al*. 2018). The Mtr4 helicase assists in RNA substrate unwinding and plays a critical role in RNA exosome processing of the 5.8S rRNA precursor (7S rRNA) (De La Cruz *et al*. 1998; Taylor *et al*. 2014). Mtr4 also acts as part of larger complexes that aid the RNA exosome in nuclear RNA quality control, including the budding yeast TRAMP (Trf4/5-Air1/2-Mtr4 polyadenylation) complex and the mammalian nuclear exosome targeting (NEXT) complex (Houseley and Tollervey 2008; Houseley and Tollervey 2009; Weir *et al*. 2010; Lubas *et al*. 2011; Stuparevic *et al*. 2013; Falk *et al*. 2014; Kilchert *et al*. 2016). Several studies that have dissected the role of Mtr4 in aiding the RNA exosome were performed in the *S. cerevisiae* system, establishing a number of *mtr4* mutations that disrupt specific interactions and functions of the helicase (Kadowaki *et al*. 1994; Kadowaki *et al*. 1995; Liang *et al*. 1996; Weir *et al*. 2010; Taylor *et al*. 2014). Thus, genetic model systems are a tractable system to investigate interactions with these nuclear cofactors that impact RNA exosome function and studies in such systems can expand our understanding of the influence that the RNA exosome can exert over various cellular processes and pathways (Cervelli and Galli 2021).

Given the variety of RNA exosome target RNAs and their link to many cellular processes, connections between the RNA exosome and human disease are not surprising. Many different human disease-linked mutations have been identified in genes encoding RNA exosome subunits (Fasken *et al*. 2020). Mutations in *DIS3*, which encodes the catalytic component of the RNA exosome in humans (Staals *et al*. 2010), are the fourth most common single nucleotide variation identified in multiple myeloma (∼10% of all newly diagnosed patients) (Chapman *et al*. 2011; Lohr *et al*. 2014). Multiple myeloma, which is a currently incurable cancer of the long-lived antibody-secreting plasma cells of the bone marrow, is the second most common hematologic malignancy accounting for 10–15% of incidence and 20% of deaths related to cancer of the blood and bone marrow (Alexander *et al*. 2007; Laubach *et al*. 2011). Multiple myeloma-associated *DIS3* mutations disrupt proper RNA degradation and processing in both mammalian cells and budding yeast mutant cells (Tomecki *et al*. 2014; Weissbach *et al*. 2015; Boyle *et al*. 2020). However, additional mechanistic studies are required to understand how mutations in *DIS3*, and the function of the RNA exosome, could contribute to pathogenesis in multiple myeloma.

Human disease mutations have also been identified in the genes encoding the non-catalytic, structural subunits of the RNA exosome. Clinical studies have linked mutations in *EXOSC* genes to various, tissue-specific human pathologies comprising a growing family of diseases termed “RNA exosomopathies” (Wan *et al*. 2012; Boczonadi *et al*. 2014; Eggens *et al*. 2014; Di Donato *et al*. 2016; Burns *et al*. 2017; Slavotinek *et al*. 2020; Somashekar *et al*. 2021). RNA exosomopathy mutations have been found in all three genes that encode the cap subunits (*EXOSC1/2/3*) and several ring subunit genes (*EXOSC5/8/9*), with most being missense mutations that result in single amino acid substitutions in highly conserved domains of the subunits. Most RNA exosomopathy diseases are neurological, with mutations in *EXOSC1*, *EXOSC3*, *EXOSC5*, *EXOSC8*, and *EXOSC9* causing forms of cerebellar atrophy/degeneration and neuronopathies (Wan *et al*. 2012; Boczonadi *et al*. 2014; Eggens *et al*. 2014; Burns *et al*. 2018; Slavotinek *et al*. 2020; Somashekar *et al*. 2021). In contrast, patients with RNA exosomopathy mutations in *EXOSC2* have a complex syndrome known as SHRF that is characterized by short stature, hearing loss, retinitis pigmentosa, and distinctive facies (OMIM #617763) (Di Donato *et al*. 2016). In vivo studies characterizing some of these *EXOSC* RNA exosomopathy mutations in *S. cerevisiae* and *Drosophila melanogaster* suggest that these pathogenic substitutions could differentially impact the function of the RNA exosome complex potentially through changes in RNA targeting and cofactor interactions (Fasken *et al*. 2017; Gillespie *et al*. 2017; Yang *et al*. 2019; De Amorim *et al*. 2020; Morton *et al*. 2020; Slavotinek *et al*. 2020; Sterrett *et al*. 2021). Modeling these pathogenic amino acid substitutions in the budding yeast RNA exosome is an invaluable tool as several RNA exosomopathies have a small patient population, making analysis with patient tissue samples challenging. Therefore, by utilizing the budding yeast system, we can begin elucidating the functional and molecular consequences resulting from human disease mutations in RNA exosome genes (Cervelli and Galli 2021).

In this study, we report several missense mutations in genes encoding structural subunits of the human RNA exosome that were identified from analysis of multiple myeloma patients and characterize the functional consequences of one of these mutations. For this analysis, we surveyed the ongoing longitudinal Multiple Myeloma Research Foundation (MMRF) study “Relating Clinical Outcomes in Multiple Myeloma to Personal Assessment of Genetic Profile” (CoMMpass) (ClinicalTrials.gov Identifier NCT01454297) to identify mutations in structural RNA exosome genes within multiple myeloma patients (Barwick *et al*. 2019). We focused on characterizing *EXOSC2 M40T*, a missense mutation that encodes an amino acid substitution EXOSC2 p.Met40Thr (M40T) in a highly conserved region of this cap subunit that interacts with the RNA helicase MTR4. To assess the effects of this amino acid substitution in EXOSC2 on the function of the RNA exosome, we utilized the budding yeast model system and generated a variant of the *S. cerevisiae* EXOSC2 ortholog, Rrp4, which models the patient EXOSC2 M40T substitution, Rrp4 M68T. As a comparative control within our studies, we included the Rrp4 G226D variant, which models a SHRF-linked pathogenic amino acid substitution in EXOSC2 p.Gly198Asp (Sterrett *et al*. 2021). The *rrp4-G226D* cells, corresponding to the SHRF *EXOSC2* exosomopathy mutation, have defects in RNA exosome function and are the only other budding yeast model of a disease-linked *EXOSC2* mutation (Sterrett *et al*. 2021). Our results show that the *rrp4-M68T* gene variant can replace the function of the essential *RRP4* gene. The *rrp4-M68T* and *rrp4-G226D* mutants show similar increases in specific RNA exosome target transcripts, suggesting shared defects in RNA processing. However, the *rrp4-M68T* mutant exhibits distinct negative genetic interactions with RNA exosome cofactor mutants, particularly *mtr4* mutants. A binding assay provides evidence that the M68T substitution impairs the interaction of Rrp4 with Mtr4. Combined, our results suggest that the Rrp4 M68T amino acid substitution, which models the multiple myeloma-associated substitution EXOSC2 M40T, alters RNA exosome function by impacting the essential interaction between the complex and Mtr4. These data are the first in vivo characterization of this isolated multiple myeloma-associated mutation and give insight into the critical and conserved interactions between the RNA exosome and its cofactors.

## Materials and methods

### Media and chemicals

All media were prepared by standard procedures (Adams *et al*. 1997). Unless stated otherwise, all chemicals were acquired from Fisher Scientific (Pittsburgh, PA), Sigma-Aldrich (St. Louis, MO), or United States Biological (Swampscott, MA).

### In silico protein structure predictions

The mCSM-PPI2 platform (Rodrigues *et al*. 2019) and the PyMOL viewer (The PyMOL Molecular Graphics System, version 2.0, Schrödinger, LLC) (PyMOL) were used for structural modeling. Platforms were used with the cryo-EM structure (PDB 6D6Q) of the human nuclear RNA exosome at 3.45Å resolution (Weick *et al*. 2018) and the X-ray diffraction structure (PDB 6FSZ) of the budding yeast nuclear RNA exosome at 4.60Å (Schuller Jan *et al*. 2018). The ConSurf server (Ashkenazy *et al*. 2010; Celniker *et al*. 2013; Ashkenazy *et al*. 2016) was used to assess the evolutionary conservation of the structure of both EXOSC2 and Rrp4.

### *Saccharomyces cerevisiae* strains and plasmids

All DNA manipulations were performed according to standard procedures (Sambrook *et al*. 1989). *Saccharomyces cerevisiae* strains and plasmids used in this study are listed in Supplementary Table 1. The *rrp4Δ* (yAV1103), *rrp4Δ mpp6Δ* (ACY2471), and *rrp4Δ rrp47Δ* (ACY2474) strains have been previously described (Schaeffer *et al*. 2009; Losh 2018; Sterrett *et al*. 2021). The *RRP45-TAP* (ACY2789) strain was obtained from Horizon Discovery Biosciences Limited and was previously described (Ghaemmaghami *et al*. 2003). The *mtr4Δ* (ACY2532) strain was constructed by deletion of the genomic *MTR4* ORF in a wild-type (W303) strain harboring a (*MTR4*, *RRP4*, and *URA3*) (pAC3714) maintenance plasmid by homologous recombination using *MTR4-UTR natMX4.* This *mtr4Δ* (ACY2532) strain was then used for consecutive deletion of the genomic *RRP4* ORF to generate the *rrp4Δmtr4Δ* (ACY2536) strain as previously described (Sterrett *et al*. 2021). Construction of the untagged *RRP4* and *rrp4-G226D* plasmids (pAC3656 and pAC3659) and the 2x-Myc-tagged *RRP4* and *rrp4-G226D* plasmids (pAC3669 and pAC3672) that contain native 3′ UTRs was reported previously (Sterrett *et al*. 2021). The *rrp4-M68T LEU2 CEN6* (pAC4206) and *rrp4-M68T-2xMyc LEU2 CEN6* (pAC4207) plasmids were generated by site-directed mutagenesis of the *RRP4* (pAC3656) or *RRP4-2xMyc* (pAC3669) plasmids using oligonucleotides containing the M68T missense mutation (Fwd 5′GAAAATACGTACCGTGACCT CT**CGT**CCAGATAGGGTCATCAGTGACC 3′, Rev 5′GGTCACTGATGACCCTATCTGG**ACG**A GAGGTCACGGTACGTATTTTC 3′) and the QuikChange II Site-Directed Mutagenesis Kit (Agilent). The *mtr4-F7A-F10A* (pAC4099) plasmid was generated as described previously (Sterrett *et al*. 2021). Similarly, the other *mtr4* mutant plasmids were constructed by site-directed mutagenesis of the *MTR4 HIS CEN6* plasmid (pAC4096) with the QuikChange II Site-Directed Mutagenesis Kit (Agilent) and oligonucleotides containing the corresponding missense mutations. The oligonucleotides used to generate the *mtr4-1* plasmid (pAC4103) contain the C942Y missense mutation (Fwd 5′CAAGCAGCAGCATTATTATCA**TAC**TTTG CATTCCAAGAACGCTG 3′, Rev 5′CAGCGTTCTTGGAATGCAAA**GTA**TGATAATAATG CTGCTGCTTG 3′). The oligonucleotides used to generate the *mtr4-R349E-N352E* plasmid (pAC4100) contain the R349E and N352E missense mutations (Falk *et al*. 2014) (Fwd 5′ GGTTGACGAAAAAAG TACCTTC**GAA**GAGGAA**GAA**TTCCAAAAAGCAATGGCGTCC 3′, Rev 5′ GGACGCCATTGC TTTTTGGAA**TTC**TTCCTC**TTC**GAAGGTACTTTTTTCGTCAACC 3′). The oligonucleotides used to generate the *mtr4-R1030A* plasmid (pAC4104) contain the R1030A missense mutation (Taylor *et al*. 2014) (Fwd 5′CGTTGATCAGAATGTTCAAG**GCA**TTAGAGGAATTGGTGAAGG 3′, Rev 5′CCTTCA CCAATTCCTCTAA**TGC**CTTGAACATTCTGATCAACG 3′) and the oligonucleotides used to generate the *mtr4-E1033W* plasmid (pAC4105) contain the E1033W missense mutation (Taylor *et al*. 2014) (Fwd 5′GAATGTTCAAGAGATTAGAG**TGG**TTGGTGAAGGAGCTGGTAGAC 3′, Rev 5′GTCTACCAGCTC CTTCACCAA**CCA**CTCTAATCTCTTGAACATTC). Plasmids were confirmed through DNA sequencing.

### *Saccharomyces cerevisiae* transformations and growth assays

All *S. cerevisiae* transformations were conducted following the standard lithium acetate (LiOAc) protocol (Da *et al*. 2000). Strains were grown in liquid YEPD (1% yeast extract, 2% peptone, 2% dextrose, in distilled water) in a rotating shaker at 30°C overnight to saturation. Cultures were normalized to a concentration of OD_600_ = 0.33 in 10 mL YEPD and then incubated at 30°C for 3–8 hours depending on the severity of their growth defect. Cells were washed and resuspended to a concentration of 2 × 10^9^ cells/mL using TE/LiOAc. Single-stranded carrier DNA (5 μL; 10 mg/mL), PEG/TE/LiOAc (300 μL), and, depending on reaction purpose, desired PCR product DNA or plasmid DNA, were added to cells. The mixture was incubated at 30°C in a shaker for 30 minutes. Following this incubation, DMSO (35 μL) was added and the cells were heat shocked for 15 minutes at 42°C, washed, and plated onto selective media.

Standard plasmid shuffle assays were performed to assess the in vivo function of the *rrp4* variants as well as genetic interaction with RNA exosome cofactor mutants. The *rrp4Δ* (yAV1103) cells containing a *RRP4 URA3* maintenance plasmid and transformed with vector (pRS315) and transformed with *RRP4* (pAC3656), *rrp4-G226D* (pAC3659), *rrp4-M68T* (pAC4206), *RRP4-2xMyc* (pAC3669), or *rrp4-M68T-2xMyc* (pAC4207) plasmid were grown on Ura^−^ Leu^−^ minimal media control plates, which select for cells that contain both the *RRP4 URA3* maintenance plasmid as well as the *RRP4*/*rrp4 LEU2* plasmid, and 5-FOA Leu^−^ minimal media plates, which select for cells that lack the *RRP4 URA3* maintenance plasmid and contain only the *RRP4*/*rrp4 LEU2* plasmid. The plates were incubated at 30°C for 2–3 days and single colonies from the 5-FOA Leu^−^ minimal media plates were selected in quadruplicate and streaked onto selective Leu^−^ minimal media plates. The cells containing only the *RRP4/rrp4 LEU2* plasmid are referred to as *RRP4*, *rrp4-G226D*, or *rrp4-M68T* cells. A similar strategy was used to generate *mtr4Δ* (ACY2532) cells that contain only the *MTR4* (pAC4096) or *mtr4-1* (pAC4103) HIS3, CEN6 plasmid. The *mtr4Δ* cells transformed with *MTR4* or *mtr4-1* were grown overnight and serially diluted and spotted onto Ura^−^ His^−^ minimal media plates and 5-FOA minimal media plates, which select for cells that lack the *URA3* maintenance plasmid and contain only the *MTR4*/*mtr4 HIS3* plasmid. Single colonies of cells containing only *MTR4* or *mtr4-1 HIS3* plasmid were collected in quadruplicate and are referred to as *MTR4* or as *mtr4-1* cells.

The in vivo function of the *rrp4-M68T* variant was assessed in growth assays on solid media and in liquid culture. For growth on solid media, *rrp4Δ* (yAV1103) cells containing only *RRP4* (pAC3656), *rrp4-G226D* (pAC3659), or *rrp4-M68T* (pAC4206) were grown in 2 mL Leu^−^ minimal media overnight at 30°C to saturation. Cell concentrations were normalized to an OD_600_ = 1, and samples were serially diluted in 10-fold dilutions and spotted onto Leu^−^ minimal media plates, Leu^−^ minimal media plates containing 25 µM fluorouracil (5-FU), YEPD plates, or YEPD plates containing 3% formamide, 150 mM hydroxyurea, or 5 µg/mL phleomycin. Plates were grown at 25°C, 30°C, and 37°C for 2–3 days. For growth in liquid culture, cells were grown in 2 mL Leu^−^ minimal media overnight at 30°C to saturation and diluted to an OD_600_ = 0.05 in Leu^−^ minimal media in a 24-well plate and growth at 37°C was monitored and recorded at OD_600_ in a BioTek SynergyMx microplate reader with Gen5 v2.04 software over 36 hr. For these liquid growth assays, the cells incubate in the microplate reader for many hours before their density is within the dynamic range of the machine to record the doubling times. For the results shown, each sample was performed in at least three independent biological replicates with three technical replicates for each biological sample. Doubling times were calculated using GraphPad Prism version 9.3.1 for Windows (www.graphpad.com), GraphPad Software, San Diego, California, USA.

### Immunoblotting

To analyze protein expression levels of C-terminally Myc-tagged Rrp4 and Rrp4 M68T, *rrp4Δ* (yAV1103) cells expressing only Rrp4-2xMyc (pAC3669) or Rrp4-M68T-2xMyc (pAC4207) were incubated in 2 mL of Leu^−^ minimal medium at 30°C and grown to saturation overnight. The 10 mL cultures with an OD_600_ = 0.2 were prepared and incubated at 30°C or 37°C for 5 hr. Yeast cell pellets were collected by centrifugation and transferred to 2 mL screw-cap tubes. Cell pellets were flash frozen with liquid nitrogen and stored at −20°C. Yeast cell lysate was prepared by resuspending pellets in 0.3 mL of RIPA-2 Buffer (50 mM Tris-HCl, pH 8; 150 mM NaCl; 0.5% sodium deoxycholate; 1% NP40; 0.1% SDS) supplemented with protease inhibitors [1 mM PMSF; 3 ng/mL PLAC (pepstatin A, leupeptin, aprotinin, and chymostatin)], followed by addition of 300 µL glass beads. Lysates were placed in a Mini Bead Beater 16 Cell Disrupter (Biospec) for 6 × 1 min at 25°C with ice submersion intervals of 1 minute between rounds and then centrifuged at 4°C at 12,000 RPM for 10 min. Protein lysate concentration was determined by Pierce BCA Protein Assay Kit (Life Technologies). Whole cell lysate protein samples (40 µg) in reducing sample buffer (50 mM Tris HCl, pH 6.8; 100 mM DTT; 2% SDS; 0.1% Bromophenol Blue; 10% Glycerol) were resolved on criterion 4–20% gradient denaturing gels (Bio-Rad) and transferred to nitrocellulose membranes (Bio-Rad) and Myc-tagged Rrp4 proteins were detected with anti-Myc monoclonal antibody 9B11 (1 : 2,000; Cell Signaling). The 3-phosphoglycerate kinase (Pgk1) protein was detected using anti-Pgk1 monoclonal antibody (1 : 30,000; Invitrogen) as a loading control. For quantitation, ImageJ v1.4 software (National Institutes of Health, MD; http://rsb.info.nih.gov/ij/) was used to measure protein band areas and intensities. Protein percentages relative to Pgk1 were calculated using GraphPad Prism version 9.3.1 for Windows (www.graphpad.com), GraphPad Software, San Diego, California, USA.

### Coimmunoprecipitations

To assess association of Rrp4 M68T with the RNA exosome complex, we utilized *RRP45-TAP* (ACY2789) cells expressing *RRP4-2xMyc* (pAC3669), *rrp4-G226D-2xMyc* (pAC3672), or *rrp4-M68T-2xMyc* (pAC4207) and immunoprecipitated Rrp45-TAP using the IgG Sepharose beads as previously described (Sterrett *et al*. 2021). Briefly, cell samples were grown in 2 mL Leu^−^ minimal media overnight at 30°C to saturation and 10–20 mL cultures with an OD_600_ = 0.2 were prepared and grown at 30°C for 5 hr. Yeast cell lysates were prepared by resuspending cell pellets in 0.5–0.75 mL of IPP150 Buffer (10 mM Tris-HCl, pH 8; 150 mM NaCl; 0.1% NP40, PMSF) supplemented with protease inhibitors [1 mM PMSF; Pierce Protease Inhibitors (Thermo Fisher Scientific)] and 300 µL of glass beads. Cells were disrupted in a Mini Bead Beater 16 Cell Disrupter (Biospec) for 4–5 × 1 min at 25°C with 1 min on ice between repetitions. Crude lysate was transferred to a chilled microcentrifuge tube and remaining beads were washed with an additional 150 µL of IPP150 Buffer. Lysate was then cleared by centrifugation at 16,000 × *g* for 10 min at 4°C. Protein lysate concentration was determined by Pierce BCA Protein Assay Kit (Life Technologies). For input samples, 40 µg of cleared lysate was collected and frozen at −20°C. For coimmunoprecipitations, 1 mg of cleared lysate in IPP150 Buffer was prepared, 30 µL of a 1 : 1 bead slurry of IgG Sepharose 6 Fast Flow Beads (GE Healthcare) was added, and samples were incubated at 4°C overnight with mixing. After overnight incubation, beads were washed three times in 1 mL IPP150 Buffer for 5 min each (IgG Sepharose beads). Whole cell lysate input samples (40 µg) and total bound samples in reducing sample buffer were boiled for 5 min at 100°C, resolved on 4–20% Criterion TGX precast polyacrylamide gels (Bio-Rad), transferred to nitrocellulose membranes (Bio-Rad). Levels of associated Rrp4-Myc proteins with the Rrp45-TAP-tagged subunit were detected by immunoblotting. Myc-tagged Rrp4 proteins were detected with mouse anti-Myc monoclonal antibody 9B11 (1 : 2,000; Cell Signaling). TAP-tagged Rrp45 protein was detected with peroxidase–antiperoxidase (PAP) soluble complex antibody produced in rabbit (1 : 5,000, Sigma-Aldrich). The 3-phosphoglycerate kinase (Pgk1) protein was detected using anti-Pgk1 monoclonal antibody (1 : 30,000; Invitrogen) as a loading control.

To assess the association of Mtr4 with Rrp4 M68T, we utilized *rrp4Δ* cells expressing Rrp4-2xMyc (pAC3669) or Rrp4-M68T-2xMyc (pAC4207). These cells were transformed with either an empty vector plasmid (pAC1) or a plasmid to express 2x-FLAG-tagged Mtr4 (pAC3719). Briefly, cell samples were grown in 2 mL His^−^Leu^−^ minimal media overnight at 30°C to saturation and 40 mL cultures with an OD_600_ = 0.2 were prepared and grown at 30°C for 4 hr. Yeast cell lysates were prepared as described above. Cleared protein lysate concentration was determined by Pierce BCA Protein Assay Kit (Life Technologies). For input samples, 50 µg of cleared lysate was collected and frozen at −20°C. For coimmunoprecipitations, 1.75 mg of cleared lysate in IPP150 Buffer was prepared, 15 µL of a 1 : 1 bead slurry of Pierce Anti-c-Myc Magnetic Beads (ThermoFisher) was added, and samples were incubated at 4°C overnight with mixing. After overnight incubation, beads were washed three times in 1 mL IPP150 Buffer for 15 sec each. Whole cell lysate input samples (50 µg) and total bound samples in reducing sample buffer were boiled for 10 min at 100°C, resolved on 4–20% Criterion TGX precast polyacrylamide gels (Bio-Rad), and transferred to nitrocellulose membranes (Bio-Rad). Levels of Mtr4-FLAG protein associated with Rrp4-Myc or Rrp4 M68T-Myc-tagged subunit were detected by immunoblotting. Myc-tagged Rrp4 proteins were detected with mouse anti-Myc monoclonal antibody 9B11 (1 : 2,000; Cell Signaling). FLAG-tagged Mtr4 protein was detected with anti-FLAG monoclonal antibody 9A3 (1 : 1,000; Cell Signaling). The 3-phosphoglycerate kinase (Pgk1) protein was detected using anti-Pgk1 monoclonal antibody (1 : 30,000; Invitrogen) as a loading control. A peroxidase AffiniPure Goat Anti-Mouse IgG, light chain specific secondary was used to detect bound proteins < 70 kDa in size. Quantitation was performed first by standardizing detected FLAG-tagged and Myc-tagged protein levels to Pgk1 levels. Then, protein levels from bound samples were normalized to unbound levels to generate a value for %Bound/Input normalized to Pgk1.

### Genetic interaction analysis

To test genetic interactions between *rrp4-M68T* and RNA exosome cofactor/subunit deletion mutants, *rrp4Δ mpp6Δ* (ACY2471) and *rrp4Δ rrp47Δ* (ACY2474) cells containing only *RRP4* (pAC3656), *rrp4-G226D* (pAC3659), or *rrp4-M68T* (pAC4206) were grown in 2 mL Leu^−^ minimal media overnight at 30°C to saturation, serially diluted, and spotted on Leu^−^ minimal media plates. The plates were incubated at 30°C or 37°C for 3 days. Cells were also grown in liquid culture as described in *S. cerevisiae* transformation and growth assay method. The *rrp4Δ mpp6Δ* (ACY2471) cells containing only *RRP4* (pAC3656), *rrp4-G226D* (pAC3659), or *rrp4-M68T* (pAC4206) were further assayed by being serially spotted onto Leu^−^ minimal media plates containing 25 µM 5-FU, YEPD plates, or YEPD plates containing 3% formamide.

To test for genetic interactions between *rrp4-M68T* and *mtr4* mutants, *mtr4-F7A-F10A*, *mtr4-1*, *mtr4-R1030A*, and *mtr4-E1033W*, *rrp4Δ mtr4Δ* (ACY2536) cells containing the (*MTR4*, *RRP4*, and *URA3*) (pAC3714) maintenance plasmid were transformed with *RRP4* (pAC3656), *rrp4-G226D* (pAC3659), or *rrp4-M68T* (pAC4206) *LEU2* plasmid and selected on Ura^−^Leu^−^ minimal media plates. Transformed cells containing both the *URA3* maintenance plasmid and the *RRP4*/*rrp4* variant plasmid were subsequently transformed with *MTR4* (pAC4096), *mtr4-F7A-F10A* (pAC4099), *mtr4-1* (pAC4103), *mtr4-R1030A* (pAC4104), or *mtr4-E1033W* (pAC4105) *HIS3* plasmid and selected on Ura^−^Leu^−^His^−^ minimal media plates. The transformed cells were then streaked to onto 5-FOA Leu^−^ His^−^ plates to select for cells that did not contain the *URA3* maintenance plasmid. The resulting *rrp4Δ mtr4Δ* cells containing only *RRP4*, *rrp4-G226D*, or *rrp4-M68T LEU2* plasmid and *MTR4* or *mtr4* variant *HIS3* plasmid were grown in 2 mL Leu^−^ His^−^ minimal media overnight at 30°C to saturation, serially diluted, and spotted on Leu^−^ His^−^ minimal media plates. The plates were incubated at 30°C and 37°C for 3 days. Cell growth was quantified on a scale from 0 to 5 across triplicate assays, with a score of “0” representing no growth detected and a score of “5” representing full growth across all dilutions analyzed. Scores were averaged and displayed as a heatmap using GraphPad Prism version 9.3.1 for Windows (www.graphpad.com), GraphPad Software, San Diego, California, USA.

### Total RNA isolation

Total RNA from *RRP4*, *rrp4-G226D*, *rrp4-M68T*, *MTR4*, or *mtr4-1* cells was isolated using the MasterPure Yeast RNA Purification Kit (Epicentre, Lucigen). Cells were incubated in 2 mL of Leu^−^ minimal medium at 30°C and grown to saturation overnight. Cultures were diluted in 10 mL to an OD_600_ = 0.2 and further incubated at 37°C for 5 hours. Cells were pelleted by centrifugation, transferred to RNase-free microcentrifuge tubes, and flash frozen with liquid nitrogen. Frozen cell pellets were stored at −80°C. RNA isolation was performed according to the MasterPure Yeast RNA Purification Kit (Epicentre, Lucigen) manufacturer's protocol. Total RNA was resuspended in 50 µL DEPC-treated water and stored at −80°C.

### RT-qPCR

All oligonucleotides used in this study are shown in Supplementary Table 2. For analysis of steady-state RNA levels using quantitative PCR, three independent biological replicates of *RRP4*, *rrp4*-*G226D*, *rrp4-M68T*, *mtr4-1*, and *MTR4* cells were grown in 2 mL Leu^−^ or His^−^ minimal media overnight at 30°C. Cultures (10 mL) with an OD_600_ = 0.2 were prepared from the saturated cultures and cells were grown at 37°C for 5 hr. Total RNA was isolated from cell pellets as described and 1 μg of total RNA was reverse transcribed to first-strand cDNA using the M-MLV Reverse Transcriptase (Invitrogen) according to the manufacturer's protocol. Quantitative PCR was performed on technical triplicates of cDNA (10 ng) from three independent biological replicates using gene-specific primers (0.5 mM; Supplementary Table 2), QuantiTect SYBR Green PCR master mix (Qiagen) on a StepOnePlus Real-Time PCR machine (Applied Biosystems; Tanneal = 55°C, 44 cycles). *ALG9* or *PGK1* was used as an internal control. The mean RNA levels were calculated by the ΔΔCt method (Livak and Schmittgen 2001). Statistical analysis comparing the control cells (*RRP4* or *MTR4*) and the mutant cells (*rrp4* or *mtr4-1*) was performed by *t*-test (α < 0.05) using GraphPad Prism version 9.3.1 for Windows (www.graphpad.com), GraphPad Software, San Diego, California, USA.

## Results

### EXOSC2 p.Met40Thr substitution is located within a conserved region of the cap subunit that interacts with the essential helicase MTR4

Mutations in the gene *DIS3*, which encodes the catalytic component of the RNA exosome, are commonly found in patients diagnosed with multiple myeloma (Chapman *et al*. 2011; Lohr *et al*. 2014), suggesting a link between RNA exosome function and disease pathology. We therefore considered whether mutations in the other components of the RNA exosome would be found in multiple myeloma patients. Missense mutations in *EXOSC* genes, which encode the structural subunits of the RNA exosome, were identified in multiple myeloma patients through interrogating the ongoing longitudinal MMRF study “CoMMpass” (ClinicalTrials.gov Identifier NCT01454297). A total of 1,154 newly diagnosed multiple myeloma patients were enrolled in CoMMpass and profiled by genomic testing and tissue sampling throughout treatment. The molecular profiling collected through CoMMpass reveals several rare missense mutations within *EXOSC* genes (Supplementary Fig. 1). One patient missense mutation identified within exon 1 of *EXOSC2* encodes EXOSC2 p.Met40Thr (M40T), which is located in a highly conserved region of the N-terminal domain of EXOSC2 (Fig. 1c). Notably, EXOSC2 Met40 lies within a key binding interface between the human RNA exosome and the RNA helicase MTR4 (Weick *et al*. 2018).

The patient with the *EXOSC2 M40T* mutation also had chromosomal aberrations including a chromosomal translocation t(11; 14) and hyperdiploidy disease. The chromosomal translocation t(11; 14) is an IgH translocation, which is an initiating event that occurs frequently in multiple myeloma (∼15–20% of patients) (Bergsagel *et al*. 1997). From the CoMMpass dataset, we determined that the variant allele frequency is 0.2266; however, the copy number of the chromosome 9 *EXOSC2* locus is 2.6, suggesting that this *EXOSC2* allele is found on the extra copy of ch9 that is present in over half the cells in the patient. Based on these findings, we conclude that this hyperdiploidy of chromosome 9 occurred after the t(11; 14) translocation event and that the *EXOSC2* mutation either cooccurred with the chromosomal gain or shortly after.

**Fig. 2.**
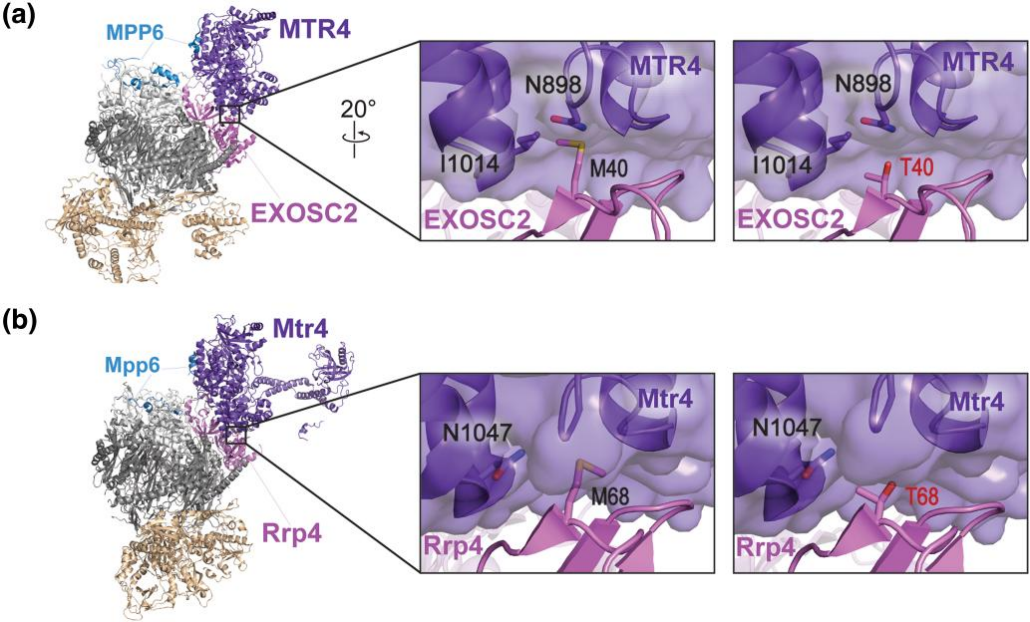
Modeling the multiple myeloma EXOSC2 M40T amino acid substitution in the human EXOSC2 cap subunit and the *S. cerevisiae* ortholog Rrp4. a) Structural modeling of the human EXOSC2 p.Met40Thr (M40T) amino acid substitution identified in a patient with multiple myeloma (PDB 6D6R) (Weick *et al*. 2018). The full structure of the human RNA exosome with the associated cofactor MTR4 (purple, labeled "MTR4") is depicted with a zoomed-in representation of the interface between EXOSC2 (pink, labeled "EXOSC2") and MTR4. Modeling of the native EXOSC2 Met40 (M40) residue (left) or the multiple myeloma-associated EXOSC2 Thr40 (T40) residue (right) is shown. The EXOSC2 Met40 residue is located in the N-terminal domain of EXOSC2, within a conserved aliphatic interface with MTR4. EXOSC2 Met40 and MTR4 associate through hydrophobic interactions, which include contacts with MTR4 Ile1014 (I1014). b) Structural modeling of the budding yeast Rrp4 Met68Thr (M68T) amino acid change, corresponding to EXOSC2 p.Met40Thr, in the budding yeast RNA exosome (PDB 6FSZ) (Schuller *et al*. 2018). The full structure of the budding yeast RNA exosome complex with the associated MTR4 ortholog, Mtr4 (purple, labeled "Mtr4"), is depicted on the left. A zoomed-in representation of the interface between Rrp4 (pink, labeled "Rrp4") and Mtr4 are shown, modeling the native Rrp4 Met68 (M68) residue (left) or the modeled multiple myeloma-associated substitution Rrp4 Thr68 (T68) residue (right). The Rrp4 Met68 residue is conserved between human and yeast and is located in the N-terminal domain of Rrp4. Similar to EXOSC2 Met40, Rrp4 Met68 associates with the helicase Mtr4 through primarily hydrophobic interactions, including contacts with several glycine residues in a neighboring loop of Mtr4. Parts of the human nuclear cofactor protein MPP6/MPH6 (blue, labeled "MPP6") and the budding yeast ortholog Mpp6 (blue, labeled "Mpp6") are also resolved in the structures shown in a) and b). Both MPP6/MPH6 and Mpp6 aid in stabilizing the interaction of the RNA helicase with the RNA exosome in addition to the direct interface made between EXOSC2 and MTR4 in humans or Rrp4 and Mtr4 in budding yeast (Falk *et al*. 2017; Wasmuth *et al*. 2017).

To explore how EXOSC2 M40T could alter the function of the RNA exosome complex, we modeled the EXOSC2 M40T amino acid substitution using a recent structure of the human RNA exosome in complex with the essential RNA helicase MTR4 (Weick *et al*. 2018). MTR4 makes several direct contacts with the RNA exosome, forming a binding interface with a total surface area of 1,440Å^2^ (Weick *et al*. 2018). Among the direct contacts between MTR4 and the RNA exosome complex, the N-terminal domain of EXOSC2 interacts with MTR4 through an aliphatic surface that includes Met40. As shown in Fig. 2a, EXOSC2 Met40 engages with a hydrophobic pocket of MTR4 including Ile1014. An amino acid substitution of Thr40, while unlikely to disrupt the aliphatic surface, could disrupt the hydrophobic interaction at this contact given the polar, shortened side chain of threonine (Fig. 2a). The EXOSC2 M40T substitution could therefore destabilize the interface between the N-terminal domain of EXOSC2 and MTR4. We also modeled the amino acid substitution in the budding yeast EXOSC2 ortholog, Rrp4, using a recent structure of the *S. cerevisiae* RNA exosome (Schuller *et al*. 2018). As shown in Fig. 2b, the budding yeast RNA exosome in complex with Mtr4 shows structural similarities to the human complex, with Rrp4 interacting directly with Mtr4. Rrp4 Met68, corresponding to the EXOSC2 Met40 residue, engages with the helicase directly through hydrophobic interactions at the binding interface. This binding interface of yeast Mtr4 is part of a large hydrophobic pocket which includes Phe924 and Ile923 (not labeled), similar to hydrophobic pocket in the human MTR4 helicase. Introduction of Thr40 would most likely disrupt this contract, similar to our predictions for the M40T substitution in EXOSC2. Furthermore, the region surrounding Rrp4 Met68 is structurally synonymous to the aliphatic surface surrounding EXOSC2 Met40, allowing for us to assess the effects of the EXOSC2 M40T amino acid substitution at the conserved interface within the yeast system.

We further explored the conservation of the binding interface of human EXOSC2 as compared to budding yeast Rrp4 using the bioinformatics tool ConSurf (Supplementary Fig. 2). The ConSurf server estimates the evolutionary conservation of amino acids of a protein based on phylogenetic trees between homologous sequences, providing conservation rates for each residue that reflect both functional and structural importance (Ashkenazy *et al*. 2010; Celniker *et al*. 2013; Ashkenazy *et al*. 2016). Consistent with the sequence alignment (Fig. 1c) and structural modeling, this tool predicts high conservation for both EXOSC2 Met40 and Rrp4 Met68 and surrounding residues (Supplementary Fig. 2). Additionally, ConSurf estimates high conservation rates at each site of contact between EXOSC2 and Rrp4 with the helicase MTR/Mtr4, further supporting the evolutionary importance of this interaction.

### *Saccharomyces cerevisiae rrp4-M68T* mutant cells that model the multiple myeloma *EXOSC2 M40T* variant show sensitivity on drugs that impact RNA metabolism

**Fig. 3.**
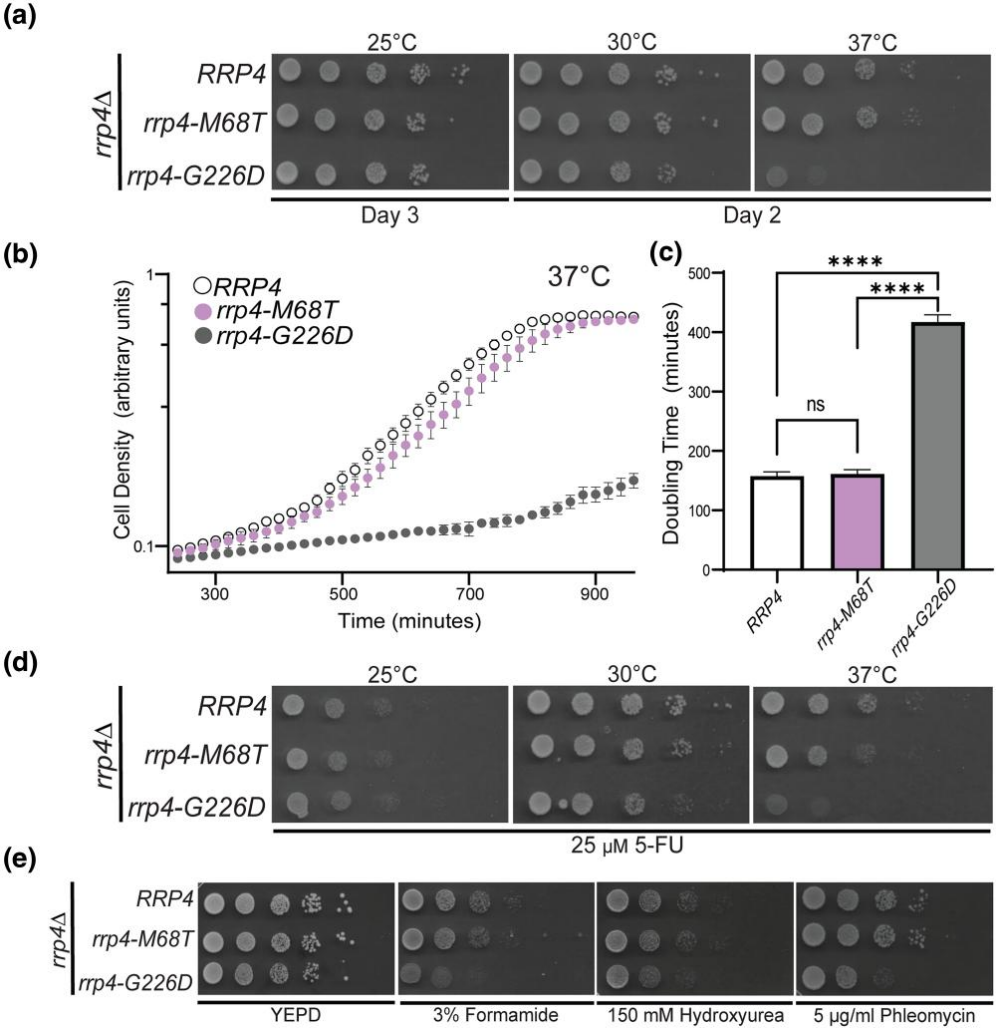
*S. cerevisiae rrp4-M68T* mutant cells that model the EXOSC2 M40T variant identified in multiple myeloma patients show impaired function on drugs that impact RNA processing. *S. cerevisiae* cells expressing Rrp4 variants that model the multiple myeloma amino acid change or, as a control, the previously characterized (Sterrett *et al*. 2021) SHRF-linked amino acid change found in EXOSC2 were generated as described in *Materials and Methods*. a–b) The *rrp4Δ* cells expressing only *RRP4* or mutant *rrp4* were serially diluted, spotted onto solid selective media grown at the indicated temperatures, or grown in liquid media at 37°C with optical density measurement used to assess cell density over time. The doubling time of these cells grown in liquid media is quantified and graphed in c). d) The *rrp4Δ* cells expressing either *RRP4* or *rrp4-M68T* were serially diluted, spotted onto solid selective media containing 25 µM fluorouracil (5-FU), and grown at the indicated temperatures. Images shown are after 2 days of growth. e) The *rrp4Δ* cells expressing only *RRP4* or *rrp4-M68T* were serially diluted and spotted onto solid YEPD media containing 3% formamide, 150 mM hydroxyurea, or 5 µg/ml phleomycin and grown at 30°C. Images shown are after two days of growth. In all assays performed, *rrp4-G226D* cells, previously reported to be severely impaired at 37°C, were included as a control (Sterrett *et al*. 2021). Data shown are representative of three independent experiments (*n* = 3).

To assess the functional consequences of the EXOSC2 M40T amino acid substitution, we generated the corresponding amino change in the *S. cerevisiae* ortholog Rrp4, M68T (Fig. 1c). We first performed a plasmid shuffle growth assay in which cells deleted for the genomic copy of *RRP4* are transformed with plasmids containing different *rrp4* alleles (see *Materials and Methods)*. This approach ensures that the background for all variants that are compared to one another is identical (Sikorski and Hieter 1989). This growth assay reveals that the *rrp4-M68T* allele can replace the essential *RRP4* gene as the *rrp4-M68T* cells grow similarly to control cells expressing wild-type *RRP4* at all temperatures examined (Fig. 3a). As a comparison, we included cells expressing the *rrp4-G226D* allele as the sole copy of the essential *RRP4* gene. The *rrp4-G226D* mutant allele models a known SHRF pathogenic amino acid change that has been shown to cause impaired RNA exosome function in vivo (Sterrett *et al*. 2021). As previously reported, cells expressing only *rrp4-G226D* show impaired growth at 37°C (Fig. 3a). Furthermore, we assessed the growth of both the *rrp4-M68T* and *rrp4-G226D* mutant cells using a liquid growth assay and quantified doubling times (Fig. 3b, 3c). These data confirm that the growth of *rrp4-M68T* cells does not differ significantly from wild-type *RRP4* cells.

To explore whether the *rrp4-M68T* mutation sensitizes cells to altered RNA processing, we tested for growth defects when cells are grown on media containing 5-fluorouracil (5-FU) (Giaever *et al*. 2004; Hoskins and Scott Butler 2007) (Fig. 3d). The *rrp4-M68T* cells show a slight growth defect compared to wild-type *RRP4* cells at 30°C when grown on solid media containing 25 µM 5-FU. This growth defect is more evident when the cells are challenged with both 37°C and 25 µM 5-FU. As a comparison, the *rrp4-G226D* cells show a severe growth defect when grown on solid media containing 5-FU both at 30°C and 37°C. To further explore whether the *rrp4-M68T* cells exhibit other changes in cell growth, we tested for growth defects when cells are grown on media containing chemicals that disrupt different cellular pathways (Fig. 3e). Formamide alters RNA metabolism (Hoyos-Manchado *et al*. 2017), hydroxyurea impairs DNA synthesis (Slater 1973), and phleomycin acts as a mutagen by introducing double-strand breaks in DNA (Suzuki *et al*. 1970). The *rrp4-M68T* cells do not show any increased sensitivity when grown at 30°C on solid media containing 3% formamide, 150 mM hydroxyurea, or 5 µg/mL phleomycin (Fig. 3e). In contrast, the *rrp4-G226D* cells show enhanced growth defects at 30°C when grown on solid media containing 3% formamide, 150 mM hydroxyurea, or 5 µg/mL phleomycin. Taken together, these data suggest that the *rrp4-M68T* cells are sensitive to defects in RNA processing but do not exhibit the same extent of disrupted cellular pathways as the previously studied *rrp4-G226D* cells, which model a pathogenic RNA exosomopathy mutation that has severely impaired RNA exosome function in vivo (Sterrett *et al*. 2021).

### *rrp4-M68T* cells have impaired RNA exosome function in processing RNA targets linked to Mtr4-RNA exosome interactions

**Fig. 4.**
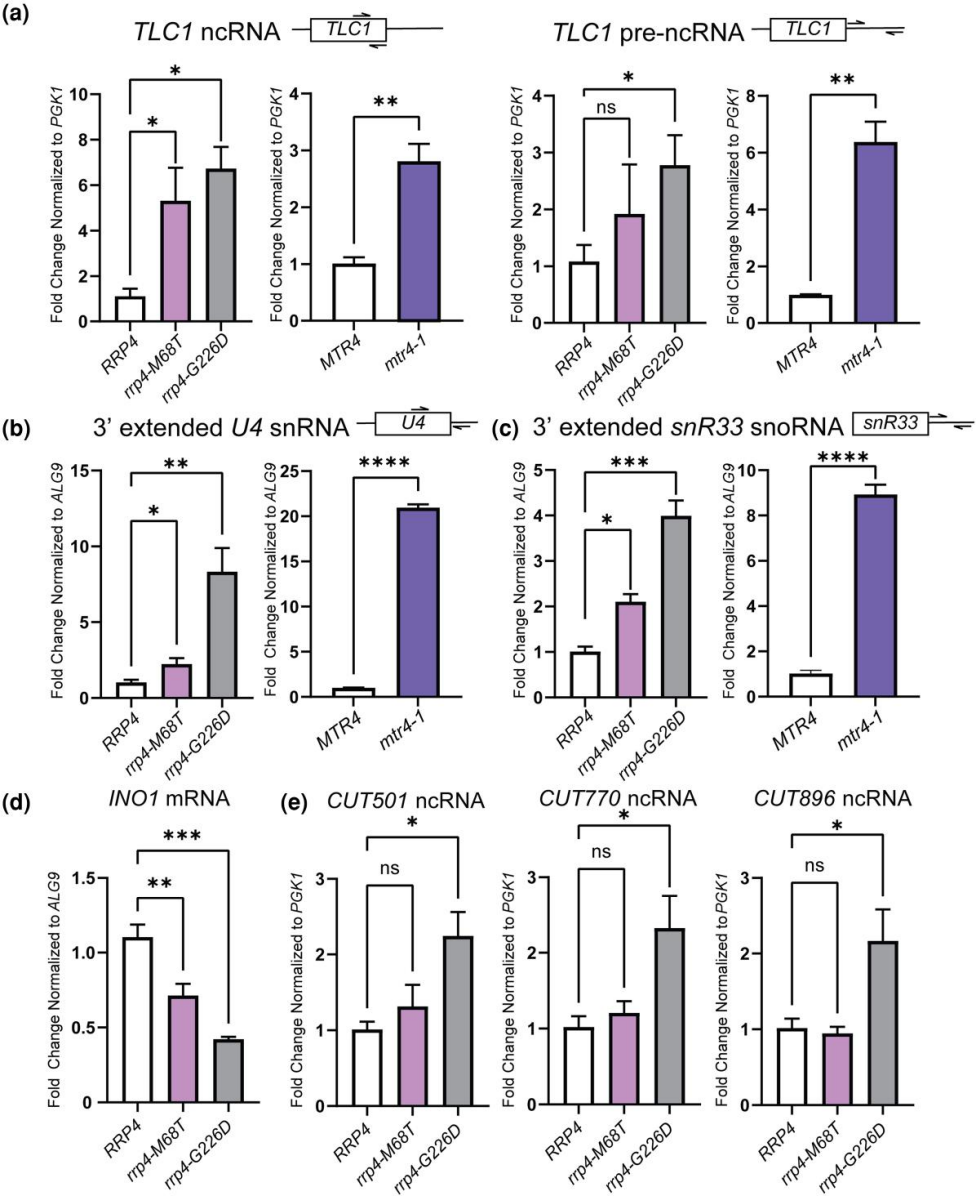
The *rrp4-M68T* mutant cells show elevated levels of specific RNA exosome target transcripts that depend on the Mtr4-RNA exosome interaction in vivo. The steady-state level of several RNA exosome target transcripts was assessed in *rrp4-M68T* cells (denoted in pink). The steady-state levels of these RNAs were also assessed in the previously described *rrp4* variant *rrp4-G226D* as a control (denoted in gray). a) The *rrp4-M68T* cells show an elevated steady-state level of mature *TLC1* telomerase component ncRNA relative to *RRP4* cells. The steady-state level of the precursor *TLC1* ncRNA in *rrp4-M68T* cells follows this upward trend though not statistically significant compared to *RRP4* cells. This increase in both mature and precursor *TLC1* is also observed in *mtr4-1* (denoted in purple) compared to the wild-type control *MTR4*. b) The *rrp4-M68T* cells show an elevated steady-state level of 3′-extended pre-*U4* snRNA relative to *RRP4* cells. The *rrp4-G226D* mutant cells and *mtr4-1* cells have even higher steady-state levels of this pre-*U4* snRNA when compared to the *RRP4* and *MTR4* controls, respectively. c) The *rrp4-M68T* cells show an elevated steady-state level of 3′ extended *snR33* snoRNA relative to *RRP4* cells that is similar to the increase observed in the *rrp4-G226D* mutant cells and *mtr4-1* cells. d) The *rrp4-M68T* cells show a decreased steady-state level of the mRNA transcript *INO1* compared to wild-type *RRP4* control cells. A decrease in this mRNA was shown previously in *rrp4-G226D* cells (Sterrett *et al*. 2021). e) The steady-state levels of non-coding, CUTs, *CUT501*, *CUT770*, and *CUT896*, are not significantly increased in *rrp4-M68T* cells compared to control as shown previously in the *rrp4-G226D* mutant cells (Sterrett *et al*. 2021). In a–e), total RNA was isolated from cells grown at 37°C and transcript levels were measured by RT-qPCR using gene-specific primers and graphed as described in *Materials and Methods.* Gene-specific primer sequences are summarized in Supplementary Table 2. The location of primers specific to the ncRNA transcripts are graphically represented by the cartoons above each bar graph. Within the cartoon transcript, the box represents the body of the mature transcript. Error bars represent standard error of the mean from three biological replicates. Statistical significance of the RNA levels in *rrp4* variant cells relative to *RRP4* cells and in the *mtr4*-1 cells relative to *MTR4* cells is denoted by an asterisk (*P-value ≤ 0.05, **P-value ≤ 0.01, ***P-value ≤ 0.001, and ****P-value ≤ 0.0001).

To further assess the in vivo consequences on RNA exosome function of the *rrp4-M68T* variant, we measured the steady-state levels of several RNA exosome targets in *rrp4-M68T* cells using RT-qPCR. We assessed the steady-state levels of precursor RNAs that are targeted by the RNA exosome and are impacted by *mtr4* mutant alleles, including the telomerase component RNA *TLC1*, which is processed by the RNA exosome in a manner dependent on TRAMP complex association, and the 3′ extended forms of *U4* snRNA and *snR33* snoRNA (Van Hoof *et al*. 2000; Houseley *et al*. 2006; Coy *et al*. 2013). In this analysis, we included both *rrp4-G226D* and *mtr4-1* cells as comparative controls. The *mtr4-1* cells have a missense mutation in *MTR4* that results in accumulation of polyadenylated targets within the nucleus (Kadowaki *et al*. 1994; Kadowaki *et al*. 1995; Liang *et al*. 1996; Weir *et al*. 2010). We detect increases in the steady-state level of both mature and precursor *TLC1* in *rrp4-M68T* cells similar to that observed in *rrp4-G226D* cells (Fig. 4a) (Sterrett *et al*. 2021). Both mature and precursor *TLC1* steady-state levels are significantly increased in *mtr4-1* cells. Furthermore, we detect a significant increase in the steady-state level of the 3′ extended forms of the *U4* snRNA and *snR33* snoRNA in the *rrp4-M68T* cells (Fig. 4b and 4c). This increase in the levels of the extended form of these target RNAs is also observed in the *rrp4-G226D* cells and, to an even larger extent, the *mtr4-1* cells. We also assessed steady-state levels of 5.8S rRNA precursors in *rrp4-M68T* and found no accumulation compared to wild-type *RRP4* cells (Supplementary Fig. 3), although levels of both mature 5.8S rRNA and pre-5.8S rRNA do increase in *rrp4-G226D* cells which supports previous observations that the *rrp4-G226D* cells exhibit accumulation of 7S rRNA (Sterrett *et al*. 2021).

We also measured the steady-state levels of select targets that are impacted within *rrp4-G226D* cells (Sterrett *et al*. 2021). We assessed the target *INO1*, which encodes inositol-3-phosphate synthetase (Donahue and Henry 1981; Klig and Henry 1984). *INO1* mRNA has previously been identified as a transcript bound to the catalytic subunit of the RNA exosome (Delan-Forino *et al*. 2017) and was the most significantly decreased transcript in a previous RNA-Seq analysis of the *rrp4-G226D* cells (Sterrett *et al*. 2021). In *rrp4-M68T* cells, the steady-state level of *INO1* is significantly decreased, similar to results for *rrp4-G226D* (Fig. 4d). We also assessed three CUTs that accumulate within *rrp4-G226D* cells (Sterrett *et al*. 2021). The steady-state levels of these three CUTs are not significantly increased in *rrp4-M68T* cells (Fig. 4e). Taken together, these data suggest that the *rrp4-M68T* cells have some molecular consequences resulting from the modeled multiple myeloma amino acid substitution; however, they differ from those resulting from the modeled SHRF substitution in the *rrp4-G226D* cells.

### The Rrp4 M68T variant can associate with the RNA exosome complex

**Fig. 5.**
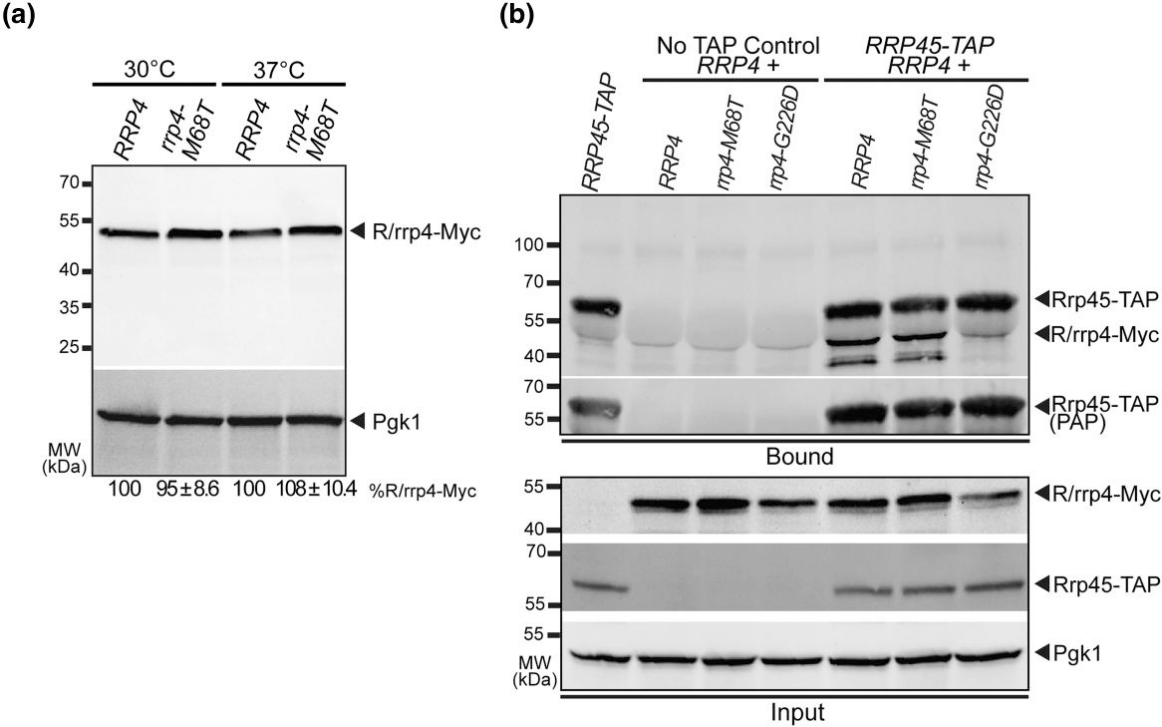
The modeled multiple myeloma amino acid substitution in Rrp4 does not impact the Rrp4 protein level or association of the cap subunit with the RNA exosome complex. a) The steady-state level of the Rrp4 M68T protein variant is equal to that of wild-type Rrp4 at both 30°C and 37°C. Lysates of *rrp4Δ* cells expressing Myc-tagged wild-type Rrp4 or Rrp4 M68T grown at 30°C or 37°C were analyzed by immunoblotting with an anti-Myc antibody. An anti-Pgk1 antibody was used to detect 3-phosphoglycerate kinase (Pgk1) as a loading control. The mean percentage of Rrp4 M68T-Myc normalized to Rrp4-Myc from four independent experiments (*n* = 4) is shown. Quantitation of the immunoblot was performed as described in *Materials and Methods*. b) The Rrp4 M68T variant coprecipitates with the RNA exosome core subunit Rrp45 in the presence of a wild-type copy of Rrp4. Tandem affinity purification-tagged Rrp45 was immunoprecipitated from *RRP45-TAP* cells expressing endogenous, wild-type Rrp4, and coexpressing Myc-tagged Rrp4, Rrp4 M68T, or, as a control, Rrp4 G226D grown at 30°C and bound (top) and input (bottom) samples were analyzed by immunoblotting. As a control, immunoprecipitations were also performed from untagged *RRP45* cells (no TAP control) expressing Myc-tagged Rrp4 and Rrp4 variants. The bound/input level of Rrp4-Myc was detected with anti-Myc antibody and the bound/input level of Rrp45-TAP was detected with a peroxidase–antiperoxidase (PAP) antibody. Bound Rrp45-TAP is also detected by the anti-Myc antibody as the protein A moiety of the TAP tag binds to the antibody. The input level of 3-phosphoglycerate kinase (Pgk1) was detected with an anti-Pgk1 antibody and shown as a loading control. Data shown is representative of three independent experiments (*n* = 3).

The sensitivity of the *rrp4-M68T* cells to drugs that impact RNA processing (Fig. 3d) and the observed accumulation of key RNA exosome target RNAs (Fig. 4) suggest that RNA exosome function may be impaired by the modeled multiple myeloma amino acid substitution. Previous studies suggest that SHRF-linked amino acid substitutions modeled in Rrp4 affect the RNA exosome function in part by disrupting complex integrity (Sterrett *et al*. 2021). To provide a first step toward assessing the impact of the modeled multiple myeloma amino acid substitution on the association of Rrp4 with other RNA exosome core subunits, we first assayed the protein level of Rrp4 M68T. We measured the steady-state level of the Myc-tagged Rrp4 M68T subunit when expressed as the sole copy of the Rrp4 protein in *rrp4Δ* cells grown at either 30°C or 37°C (Fig. 5a). Immunoblotting reveals that the steady-state level of Rrp4 M68T is comparable to wild-type Rrp4 at both temperatures tested. We next performed coimmunoprecipitations using *RRP45-TAP* cells that contain the endogenous, genomic *RRP4* gene and express a C-terminally tandem affinity purification (TAP)-tagged Rrp45 core subunit from the endogenous *RRP45* locus. We expressed Rrp4-Myc or Rrp4 M68T-Myc from plasmids in these *RRP45-TAP* cells. The Rrp45-TAP protein was immunoprecipitated and association of the Myc-tagged Rrp4 variants was assayed by immunoblotting (Fig. 5b). Under these conditions in which an endogenous copy of *RRP4* is present, we can detect association of Rrp4 M68T-Myc with Rrp45-TAP at levels equal to that of Rrp4-Myc. As a comparative control, we also performed coimmunoprecipitations with *RRP45-TAP* cells expressing an exogenous Rrp4 G226D-Myc variant. Under these conditions with an endogenous copy of *RRP4* present, we cannot detect association of Rrp4 G226D-Myc with the TAP-tagged core subunit, supporting previous observations (Sterrett *et al*. 2021). Taken together, these data suggest that Rrp4 M68T is biochemically similar to a wild-type Rrp4 subunit, and the multiple myeloma amino acid substitution likely has no impact on RNA exosome complex integrity.

### The *rrp4-M68T* mutant shows negative genetic interactions with *mtr4* mutants that impact TRAMP complex association and RNA helicase unwinding

**Fig. 6.**
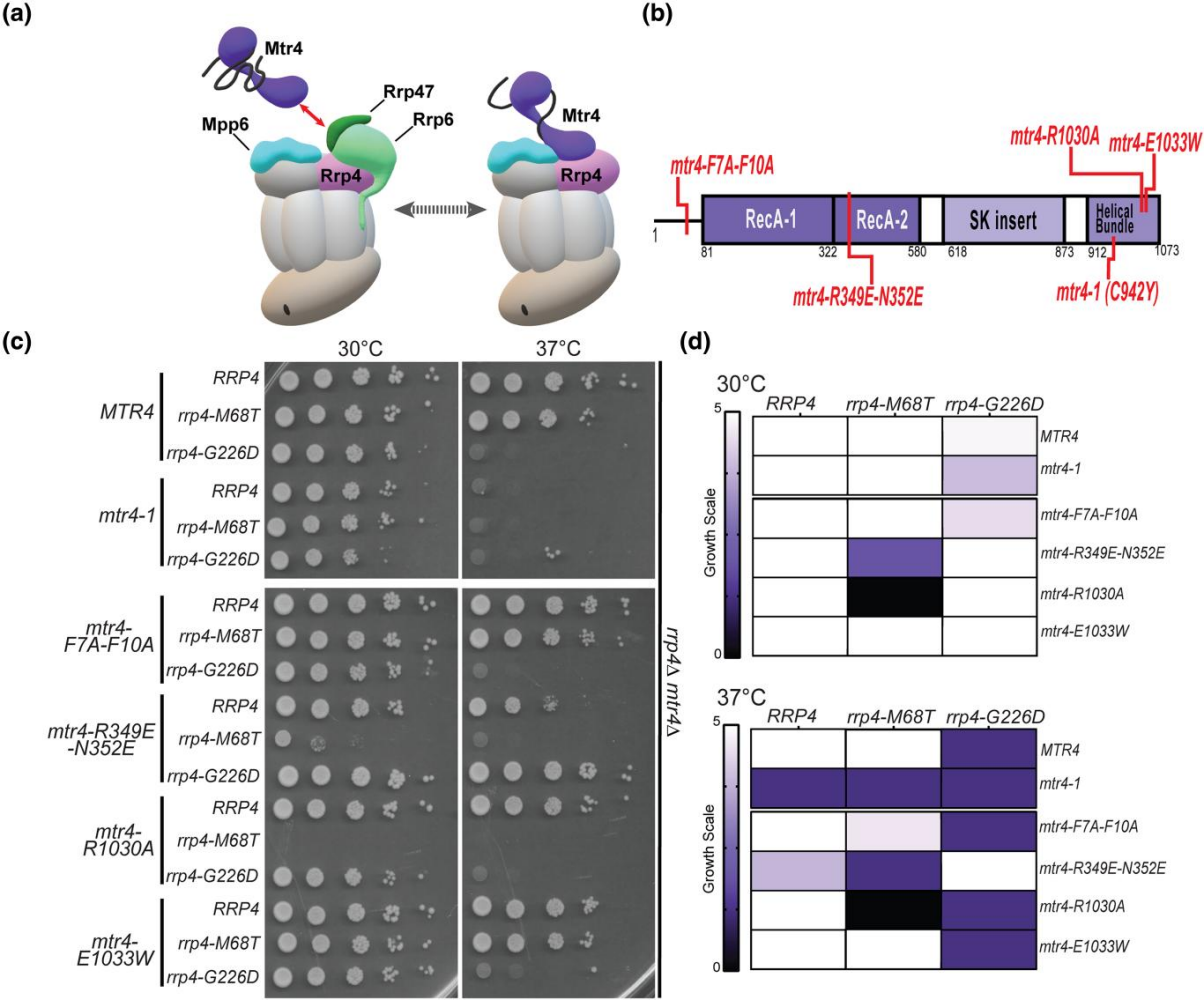
The *rrp4-M68T* mutant cells show specific negative genetic interactions with *mtr4* mutants that are predicted to impair the Trf4/5-Air1/2-Mtr4 (TRAMP) complex. a) Cartoon depicting the budding yeast nuclear RNA exosome with interacting nuclear cofactors Mpp6 (turquoise, labeled "Mpp6") and Rrp47 (dark green, labeled "Rrp47"), the exoribonuclease Rrp6 (light green, labeled "Rrp6"), and the essential RNA helicase, Mtr4 (purple) (Schuch *et al*. 2014; Falk *et al*. 2017; Schuller *et al*. 2018). The association of Mtr4 with the RNA exosome is facilitated by interactions between Mtr4 and Rrp6/Rrp47 (denoted by the red arrowed line) and by interactions with Mpp6 which is associated with the Rrp40 RNA exosome subunit and the Rrp4 subunit (Weir *et al*. 2010; Schuch *et al*. 2014; Wasmuth *et al*. 2017). The association of Mtr4 with the RNA exosome can also facilitate interaction with the Trf4/5-Air1/2-Mtr4 polyadenylation (TRAMP) complex, which triggers degradation of certain RNA targets by adding short oligo(A) tails to the 3′ end of these targets and delivering them to the RNA exosome (Houseley *et al*. 2006; Anderson and Wang 2009; Belair *et al*. 2018; Ogami *et al*. 2018). In addition to Mtr4, the TRAMP complex is composed of a noncanonical poly(A) polymerase, Trf4/5, and a zinc-knuckle RNA binding protein, Air1/2 (Belair *et al*. 2018). Central to the degradation of TRAMP-targeted RNAs by the RNA exosome is the association of Mtr4 with Trf4/5, Air1/2, and the cap subunits and nuclear cofactors of the RNA exosome complex (Falk *et al*. 2014; Schuch *et al*. 2014). b) Domain structure for *S. cerevisiae* Mtr4. The helicase has a low-complexity N-terminal sequence followed by the conserved helicase region. The helicase region is composed of two RecA domains and a helical domain (labeled helical bundle) that form the globular core typical of DExH family proteins. The helical bundle was originally described as the “ratchet” domain for its role in translocating nucleic acid by a Brownian ratchet (Büttner *et al*. 2007). In addition, Mtr4 contains an insertion domain and KOW domain that fold into a helical stalk (labeled SK insertion) (Jackson *et al*. 2010; Weir *et al*. 2010). The amino acid changes used for this experiment are labeled in red along the domain structure. c) Double mutant cells containing *rrp4-M68T* and specific *mtr4* mutants show lethality at both 30°C and 37°C. The *rrp4Δ mtr4Δ* double mutant cells were serially diluted, spotted onto solid media, and grown at the indicated temperatures for 3 days. The *mtr4* mutant plasmids included in this experiment are as follows; *mtr4-1—*a temperature-sensitive mutant that contains a missense mutation resulting in the amino acid substitution Cys942Tyr, which causes accumulation of poly(A)+ RNA in the nucleus at 37°C (Kadowaki *et al*. 1994; Kadowaki *et al*. 1995; Liang *et al*. 1996); *mtr4-F7A-F10A—*an *mtr4* allele that impairs the interaction with Rrp6/Rrp47 (Schuch *et al*. 2014); *mtr4-R349E-N352E—*a mutation that impairs the association of Mtr4 with the poly(A) RNA polymerase Trf4 with the Mtr4 helicase (Falk *et al*. 2014); *mtr4R-1030A* and *mtr4-E1033W*—two mutations within the helical bundle that differentially impact nucleic acid unwinding by Mtr4 (Taylor *et al*. 2014). *mtr4-1* mutant cells expressing *RRP4*, *rrp4-M68T*, or *rrp4-G226D* show lethality at 37°C presumably due to the known temperature-sensitive nature of the *mtr4-1* allele (Liang *et al*. 1996). Growth of double mutant cells containing *rrp4-M68T* or *rrp4-G226D* is shown. d) Summary of *rrp4 mtr4* mutant cell growth. Triplicate solid media assays were performed on double mutant cells containing *rrp4-M68T* or *rrp4-G226D* and the series of *mtr4* variants. Cell growth at both 30°C and 37°C was semiquantified on a scale of zero (no growth; black) to five (comparable to *RRP4* wild-type growth; white). Growth scale of the double mutant cells is represented through the color gradient on the two heatmaps. All double mutant cells were generated as described in *Materials and Methods*. Images shown are from a singular solid media growth assay with all samples plated on the same Leu^−^ media plate. Data are representative of three independent experiments (*n* = 3).

As the Rrp4 M68T variant associates with the RNA exosome complex and has a steady-state level equivalent to wild-type Rrp4, the observed sensitivity to disrupted RNA processing in *rrp4-M68T* cells (Fig. 3d) and accumulation of select RNA exosome target transcripts (Fig. 4) could be due to altered interaction between Rrp4 and Mtr4. As depicted in Fig. 6a, the nuclear RNA exosome cofactors Mpp6 and Rrp47 and the associated exonuclease Rrp6 aid in recruiting Mtr4 to the RNA exosome (Wasmuth *et al*. 2017). Rrp6 and Rrp47 form a composite site that binds to the N-terminus of Mtr4, recruiting the helicase to the RNA exosome (Schuch *et al*. 2014). Mtr4 forms contacts with the cap subunit Rrp4 and the cofactor Mpp6, stabilizing the helicase on the RNA exosome complex through a very conserved interface between the cap subunit and the helicase (Falk *et al*. 2017; Weick *et al*. 2018). The interaction between Mtr4 and Rrp4 provides a surface for the RNA exosome to associate with the TRAMP complex, which helps facilitates nuclear RNA surveillance and quality control of ncRNA (Lacava *et al*. 2005; Vaňáčová *et al*. 2005; Houseley and Tollervey 2008; Houseley and Tollervey 2009; Lubas *et al*. 2011; Stuparevic *et al*. 2013; Rodríguez-Galán *et al*. 2015; Kilchert *et al*. 2016; Zinder and Lima 2017; Ogami *et al*. 2018; Belair *et al*. 2019). In addition to Mtr4, the TRAMP complex is composed of a zinc-knuckle RNA binding protein, Air1 or Air2, and a non-canonical oligo(A) polymerase, Trf4 or Trf5, that oligoadenylates RNA (Belair *et al*. 2018). The TRAMP complex triggers degradation by adding short polyadenylated tails to the 3′ end of substrate RNA and delivering them to the RNA exosome (Houseley *et al*. 2006; Anderson and Wang 2009; Belair *et al*. 2018; Ogami *et al*. 2018).

To assess genetic interactions between Mtr4 and the RNA exosome in *S. cerevisiae* modeling the multiple myeloma patient mutation, we performed an analysis of a series of five *mtr4* mutant alleles that introduce amino acid substitutions in Mtr4 as summarized in Fig. 6b. We also included the *rrp4* mutant variant, *rrp4-G226D*, for comparison as this *rrp4* variant has a negative interaction with the *mtr4-F7A-F10A* mutant allele (Sterrett *et al*. 2021). Included within our five *mtr4* alleles is the *mtr4-1* allele, a known temperature sensitive mutant (Kadowaki *et al*. 1994; Kadowaki *et al*. 1995; Liang *et al*. 1996; Weir *et al*. 2010). These genetic data are shown both as representative solid growth assays (Fig. 6c) and as a heatmap (Fig. 6d), which summarizes data from three independent replicates for all these genetic experiments. As predicted, *RRP4 mtr4-1* cells have a severe growth defect at 37°C that is shared by both double mutant *rrp4-M68T mtr4-1* and *rrp4-G226D mtr4-1*.

Two of the five *mtr4* mutant alleles, *mtr4-F7A-F10A* and *mtr4-R349E-N352E*, impair protein–protein interactions of Mtr4 in *S. cerevisiae*. The Mtr4 F7A F10A variant disrupts Mtr4 interactions with Rrp6/Rrp47 by introducing two amino acid substitutions, F7A and F10A, into the N-terminus of Mtr4 (Fig. 6b) (Schuch *et al*. 2014). The *mtr4-R349E-N352E* mutant allele impairs the association of Mtr4 with the poly(A) RNA polymerase Trf4 within the TRAMP complex and thus disrupts the recruitment of the TRAMP complex to the RNA exosome (Falk *et al*. 2014). The *rrp4-M68T mtr4-F7A-F10A* double mutant cells grow similarly to *rrp4-M68T* cells at 30°C; however, at 37°C, the double mutant cells show a mild growth defect in comparison with the single mutant *rrp4-M68T* and the *RRP4 mtr4-F7A-F10A* cells (Fig. 6d). As shown previously, the *rrp4-G226D mtr4-F7A-F10A* cells show a severe growth defect at 37°C compared to *rrp4-G226D* cells (Sterrett *et al*. 2021). The *rrp4 M68T mtr4-R349E-N352E* double mutant cells show severe growth defects at 30°C and 37°C compared to the single mutant *rrp4-M68T* cells or the *RRP4 mtr4-R349E-N352E* control cells. In contrast, the *rrp4 G226D mtr4-R349E-N352E* double mutant cells show no difference in growth at 30°C compared to the *RRP4 mtr4-R349E-N352E* control cells and improved growth compared to the single mutant *rrp4-G226D* cells at 37°C (Fig. 6c and 6d).

The final two *mtr4* mutant alleles that we tested for genetic interaction with the *rrp4-M68T* variant impact nucleic acid unwinding by Mtr4 (Taylor *et al*. 2014). Studies of an RNA-bound Mtr4 structure demonstrate that residues of R1030 and E1033 mediate key nucleic acid base interactions with the helicase helical bundle (Weir *et al*. 2010; Taylor *et al*. 2014). Mutagenesis of these residues in *S. cerevisiae*, generating the mutant alleles *mtr4-R1030A* and *mtr4-E1033A*, reveals that these residues play important but distinct roles in helicase activity (Taylor *et al*. 2014). The *rrp4-M68T mtr4-R1030A* double mutant cells are not viable at either temperature tested. In contrast, the *rrp4-M68T mtr4-E1033A* double mutant cells are viable and grow similar to the *RRP4 mtr4-E1033A* control cells at both 30°C and 37°C. The growth defect of *rrp4-G226D* at 37°C is too severe to assess genetic interactions with either the *mtr4-R1030A* or the *mtr4-E1033A* mutation under these growth conditions. However, in contrast to the lethality observed for the *rrp4-M68T mtr4-R1030A* double mutant, the *rrp4-G226D mtr4-R1030A* and *rrp4-G226D mtr4-E1033A* double mutant cells have comparable growth to the single mutant *rrp4-G226D* cells as well as the *RRP4 mtr4-R1030A* and *RRP4 mtr4-E1033A* control cells at 30°C. The *rrp4-M68T* double mutants that show synthetical lethality are viable when rescued by expression of a wild-type *RRP4* plasmid (Supplementary Fig. 4), demonstrating that the growth defects and lethality observed are due to negative genetic interactions between the *rrp4* and *mtr4* mutants. Taken together, these data suggest that the *rrp4-M68T* cells have negative genetic interactions with specific *mtr4* mutant alleles, distinct from those previously described for the *rrp4-G226D* mutant model.

### The *rrp4-M68T* mutant shows negative genetic interactions with *mpp6Δ*

**Fig. 7.**
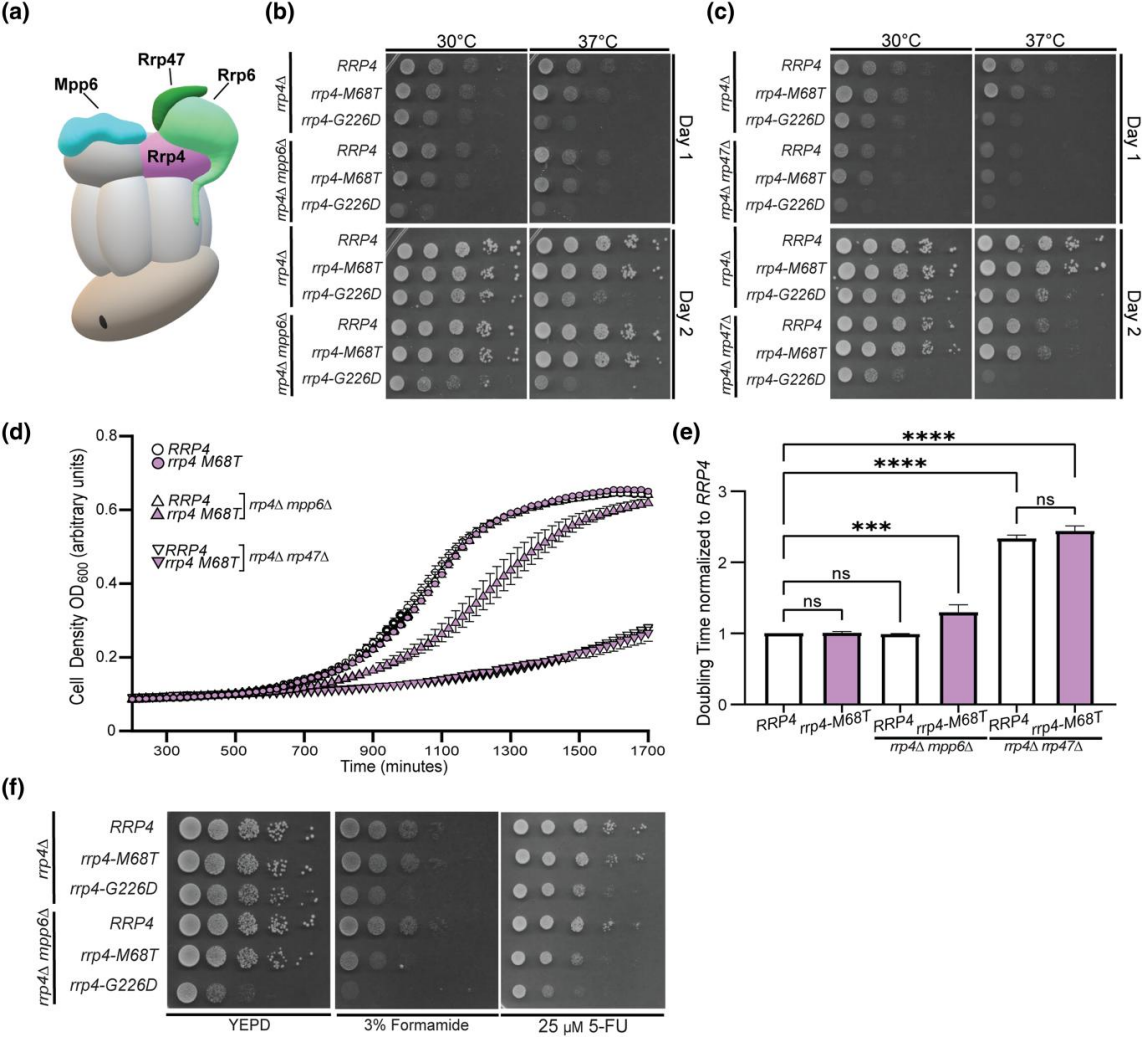
The *rrp4-M68T mpp6*Δ double mutant cells exhibit impaired growth that is exacerbated on drugs that impact RNA processing. a) Cartoon schematic of the budding yeast nuclear RNA exosome in complex with nuclear cofactors Mpp6 (turquoise, labeled "Mpp6") and Rrp6/47 (light green/dark green, labeled "Rrp6" and "Rrp47") (Schuller *et al*. 2018). Serial dilution growth assays of double mutant (B) *rrp4-M68T mpp6Δ* or (C) *rrp4-M68T rrp47Δ* cells at 30°C and 37°C. The double mutant cells (*rrp4Δ* with *mpp6Δ* or *rrp47Δ*) containing control *RRP4* or *rrp4* variants *rrp4-M68T* and *rrp4-G226D* plasmids were serially diluted, spotted onto selective solid media, and grown at the indicated temperatures for two days. The double mutant cells *rrp4-G226D mpp6Δ* and *rrp4-G226D rrp47Δ* were included as a comparative control and show growth defects as described previously (Sterrett *et al*. 2021). Data shown are representative of three independent assays (*n* = 3). d) and e) Double mutant cells containing *rrp4-G226D* and *mpp6Δ* exhibit a statistically significant increase in doubling time in liquid culture. Double mutant cells (*rrp4Δ mpp6Δ* or *rrp4Δ rrp47Δ)* containing control *RRP4* or *rrp4-M68T* plasmids were diluted in selective media and grown at 37°C with optical density measurements used to assess cell density over time. Data shown is collected from four independent samples (*n* = 4). e) Doubling time for each sample was quantified and normalized to the growth rate of control *RRP4* cells. All calculations were performed as described in *Materials and Methods.* Full liquid growth curves of both *rrp4-M68T mpp6Δ* and *rrp4-M68T rrp47Δ* mutant cells are shown in Supplementary Fig. 5. f) Double mutant cells *rrp4-M68T mpp6Δ* exhibit impaired growth on solid media containing drugs impacting RNA processing. The *rrp4Δ mpp6Δ* cells expressing *RRP4*, *rrp4-M68T*, or *rrp4-G226D* were serially diluted, spotted onto solid YEPD media containing 3% formamide or selective media containing 25 µM fluorouracil (5-FU) and grown at 30°C for three days. The *rrp4-M68T mpp6Δ* cells show impaired growth when compared to *RRP4 mpp6Δ* cells. The *rrp4-G226D mpp6Δ* cells show exacerbated growth defects on 3% formamide and 25 µM 5-FU at 30°C. Data shown are representative of three independent assays (*n* = 3).

As depicted in Fig. 6a, the nuclear RNA exosome cofactors Mpp6 and Rrp47 and the exoribonuclease Rrp6 help to recruit and stabilize the interaction with Mtr4. The exosome cofactor Rrp47 interacts with and stabilizes the exoribonuclease Rrp6 and the cofactor Mpp6 interacts with the nuclear RNA exosome through direct contacts with the cap subunit Rrp40 (Schuch *et al*. 2014; Wasmuth *et al*. 2014; Wasmuth *et al*. 2017). To further evaluate the impact that the modeled multiple myeloma amino acid substitution may have on the RNA exosome-Mtr4 interaction in vivo, we tested whether the *rrp4-M68T* variant exhibits genetic interactions with *mpp6* or *rrp47* mutants by deleting these non-essential, nuclear exosome cofactor genes *MPP6* and *RRP47* in combination with *rrp4-M68T*. For comparison, we included the *rrp4-G226D* variant as these cells have known negative genetic interactions with these mutants (Sterrett *et al*. 2021). We examined the growth of these double mutants relative to single mutants (*rrp4-M68T* or *rrp4-G226D*) and the control mutant cells (*RRP4 mpp6Δ* or *RRP4 rrp47Δ*) in solid and liquid media growth assays (Fig. 7). In the solid media growth assays, the *rrp4-M68T mpp6Δ* double mutant cells show growth very similar to the *rrp4-M68T* cells at both 30°C and 37°C after both 1 and 2 days of growth (Fig. 7b). The *rrp4-M68T rrp47Δ* cells show a severe growth defect at 37°C compared to the single mutant *rrp4-M68T*; however, this impaired growth is comparable to that of the *RRP4 rrp47Δ* cells, which has been previously reported for the single mutant *rrp47Δ* (Mitchell *et al*. 2003) (Fig. 6c). In contrast, the *rrp4-G226D mpp6Δ* and *rrp4-G226D rrp47Δ* double mutant cells show a severe growth defect at both temperatures compared to the single mutant *rrp4-G226D* cells as described previously (Sterrett *et al*. 2021).

While the solid media growth assay suggests comparable growth between the controls and the *rrp4-M68T* double mutant cells, the liquid media growth assay reveals a modest growth defect of the *rrp4-M68T mpp6Δ* at 37°C compared to both the *rrp4-M68T* and control *RRP4 mpp6Δ* cells (Fig. 7d), with the doubling time significantly longer than that of wild-type *RRP4* cells (Fig. 7e). The liquid growth assay also shows doubling times for *rrp4-M68T rrp47Δ* and *RRP4 rrp47Δ* double mutants are nearly twice that of wild-type *RRP4* cells but do not differ significantly when compared to each other (Fig. 7d and 7e). The observed growth defect of the *rrp4-M68T mpp6Δ* double mutant in liquid culture is revealed in a solid media growth assay when the cells are challenged with formamide or 5-FU (Fig. 7f). The distinct growth defect of the *rrp4-G226D mpp6Δ* double mutant is also exacerbated by growth on these chemicals. Taken together, these data suggest a negative genetic interaction between the *rrp4* variants and *mpp6* mutants, with both double mutants having exacerbated defects when challenged with drugs that impact RNA metabolism.

### The Rrp4 M68T variant shows decreased association with the essential helicase Mtr4

**Fig. 8.**
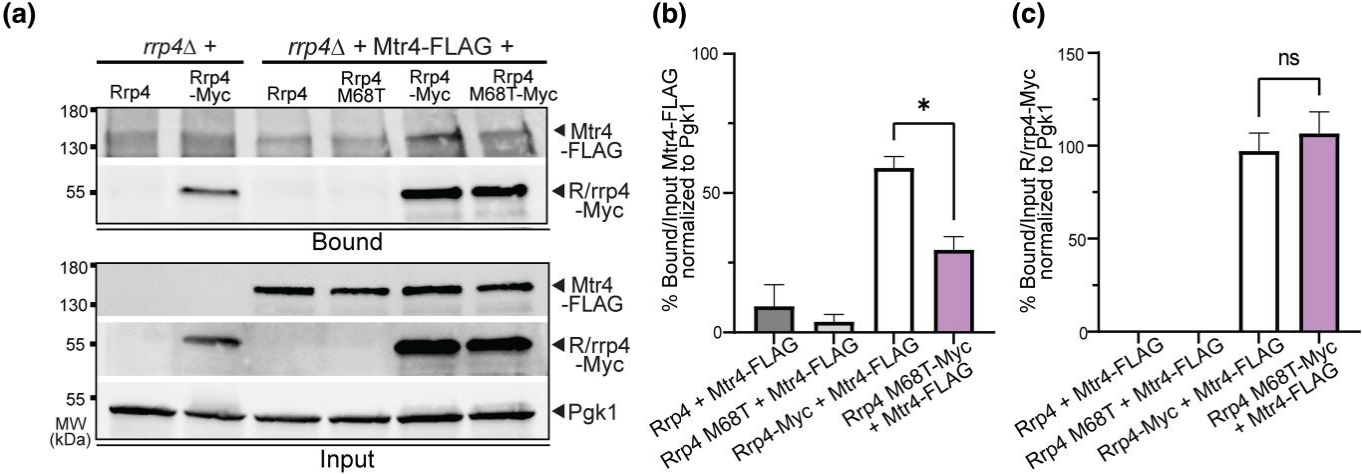
Rrp4 M68T shows decreased association with Mtr4 compared to wild-type Rrp4. a) The *rrp4Δ* cells coexpressing Rrp4-Myc variants and FLAG-tagged Mtr4 were grown at 30°C. Myc-tagged Rrp4 or Rrp4 M68T protein was immunoprecipitated from cleared lysate using anti-Myc beads and bound Mtr4-FLAG protein was detected by immunoblotting. The bound/input level of Mtr4-FLAG was detected with an anti-FLAG antibody and the bound/input level of Rrp4-Myc was detected with an anti-Myc antibody. The 3-phosphoglycerate kinase (Pgk1) serves as a loading control. b) Quantitation of the percentage of bound to input Mtr4-FLAG coimmunoprecipitated with Rrp4-Myc or Rrp4 M68T-Myc normalized to Pgk1. The graph shows the mean percentage of bound Mtr4-FLAG normalized to unbound input. Error bars represent standard error of the mean. c) Quantitation of percentage of bound to input Rrp4-Myc or Rrp4 M68T-Myc immunoprecipitated normalized to Pgk1. Error bars represent standard error of the mean. Statistical significance is denoted (*P-value ≤ 0.05; n.s. *P*-value ≥ 0.05). Data shown here collected were from two independent experiments (*n* = 2). The coimmunoprecipitations were performed and quantitated as described in *Materials and Methods*.

Given the negative genetic interactions of *rrp4-M68T* with both *mpp6* and *mtr4* mutants and the physical interactions between both EXOSC2 Met40 and Rrp4 Met68 and the RNA helicase MTR4/Mtr4 in the RNA exosome structures (Schuller *et al*. 2018; Weick *et al*. 2018), we predicted that the physical interaction between the RNA exosome and Mtr4 would be affected in *rrp4-M68T* cells. Previous studies investigated the physical interaction between Rrp4 G226D and Mtr4 using a coimmunoprecipitation assay and the data suggested that there is decreased association between the RNA exosome and the helicase in *rrp4-G226D* cells (Sterrett *et al*. 2021). We employed a similar approach to investigate whether the physical interaction between Mtr4 and the RNA exosome is impacted by the Rrp4 M68T variant. We performed a coimmunoprecipitation with cells expressing Rrp4-Myc or Rrp4 M68T-Myc as the sole copy of Rrp4 and coexpressing Mtr4-FLAG (Fig. 8). The Rrp4-Myc proteins were immunoprecipitated and association with Mtr4-FLAG was assayed by immunoblotting. As shown in Fig. 8, there is a significant decrease in the amount of Mtr4-FLAG that coimmunoprecipitates with Rrp4 M68T-Myc as compared to Rrp4-Myc (Fig. 8a-b). The amount of Rrp4 M68T-Myc and Rrp4-Myc in both the input and immunoprecipitation is comparable (Fig. 8c), showing that the difference in detected Mtr4-FLAG is not due to decreased protein levels or inefficient immunoprecipitation of Rrp4 M68T-Myc (Fig. 8c). These data, therefore, suggest that Mtr4 association with the Rrp4 cap subunit is significantly disrupted by the Rrp4 M68T amino acid substitution. Combined with the genetic data (Fig. 6 and Fig. 7), the structural modeling (Fig. 2), and the increased steady-state level of RNA exosome target RNAs (Fig. 4), these results strongly suggest that there is destabilization of the interaction between Mtr4 and the RNA exosome complex in *rrp4-M68T* cells that impacts the function of the molecular machine.

## Discussion

In this study, we modeled and analyzed a multiple myeloma patient *EXOSC2* mutation in the *S. cerevisiae* homolog *RRP4*. We generated *rrp4-M68T* mutant cells expressing the variant Rrp4 M68T, which corresponds to the EXOSC2 M40T variant. Analysis of these *rrp4-M68T* cells reveals that this amino acid substitution affects RNA exosome function. While our biochemical assays show that the Rrp4 M68T variant can associate with the RNA exosome complex and function as the sole copy of the essential Rrp4 RNA exosome cap subunit, *rrp4-M68T* cells do show growth defects when grown in media containing drugs that impact RNA processing. The *rrp4-M68T* cells also show accumulation of known RNA exosome targets. These defects in RNA exosome function could result from an impaired interaction between the complex and the essential RNA helicase Mtr4 as predicted by structural modeling. Our genetic analyses support this model as *rrp4-M68T* cells show negative genetic interactions with both *mpp6* and *mtr4* mutants. Furthermore, we demonstrate through a coimmunoprecipitation assay that the M68T substitution in Rrp4 decreases association with the Mtr4 helicase. These data suggest that the introduction of the multiple myeloma-associated amino acid change could impact the binding interface between EXOSC2 and MTR4, potentially impairing the function of the essential RNA exosome in vivo for a subset of Mtr4-dependent targets.

Structural studies reveal the evolutionary conservation of the interaction between the RNA exosome and Mtr4 (Supplementary Fig. 2), with the helicase cofactor interacting with the complex through multiple points of contact, including a direct interface with EXOSC2/Rrp4 and indirect stabilizing interactions with the cofactors Mpp6, Rrp47, and the associated exonuclease Rrp6 (Falk *et al*. 2017; Weick *et al*. 2018). This robust interaction between the complex and the essential helicase likely explains why the *rrp4-M68T* cells show no functional consequences unless challenged through introduction of drugs impacting RNA processing or loss of other stabilizing cofactors, such as in *rrp4-M68T mpp6Δ* double mutant cells. While this model would also predict a negative genetic interaction between *rrp47Δ* and *rrp4-M68T*, the *rrp4-M68T rrp47Δ* cells show a growth defect at 37°C that is indistinguishable from that of *RRP4 rrp47Δ* cells. This growth defect in *rrp4-M68T rrp47Δ* and *RRP4 rrp47Δ* cells is likely due to the loss of Rrp6 association with the RNA exosome complex given the stabilizing role that Rrp47 plays for Rrp6 (Mitchell *et al*. 2003; Wasmuth *et al*. 2014). The growth defects resulting from destabilization of Mtr4 in *rrp4-M68T rrp47Δ* cells are likely masked by the larger consequence of disassociating Rrp6 from the complex. We do detect a slight growth defect in *rrp4-M68T* cells expressing an *mtr4* variant that disrupts the stabilizing interactions between Rrp6, Rrp47, and Mtr4 (*mtr4-F7A-F10A*), pointing to the importance of the Rrp4-Mtr4 interface. We do, however, observe significant molecular consequences in the *rrp4-M68T* cells. We detect accumulation of several documented RNA exosome target transcripts, particularly those linked to RNA exosome–Mtr4 association (Van Hoof *et al*. 2000; Houseley *et al*. 2006). Using a biochemical assay, we also observe a significant decrease in interaction between Mtr4 and Rrp4 M68T as compared to wild-type Rrp4. This decreased association further suggests that the modeled multiple myeloma mutation destabilizes the interaction between the RNA exosome and the essential RNA helicase. Taken together, these data suggest that while the consequences resulting in vivo from the Rrp4 M68T variant are subtle at the macro scale, they are impactful molecularly for a specific set of target RNAs and for the biochemical interaction between the RNA exosome and Mtr4.

The interaction between the RNA exosome and Mtr4 could also be critical for other interactions, particularly those involving the TRAMP (Trf4/5-Air1/2-Mtr4 polyadenylation) complex. Our genetic analyses reveal a negative genetic interaction between *rrp4-M68T* and *mtr4-R349E-N352E*. The Mtr4 R349E N352E variant impairs Mtr4-Trf4 binding and impacts TRAMP complex assembly in vivo (Falk *et al*. 2014). The *rrp4-M68T* cells that express Mtr4 R349E N352E as the sole copy of the helicase grow very poorly at both 30°C and 37°C as compared to control *RRP4 mtr4-R349E-N352E* cells, suggesting that TRAMP complex assembly and association with the RNA exosome may also be impacted by Rrp4 M68T. Intriguingly, we also detect synthetic lethality for the *rrp4-M68T mtr4-R1030A* double mutant. This lethality is specific to *rrp4-M68T mtr4-R1030A* cells as the *rrp4-M68T* cells expressing the other helicase mutant, *mtr4-E1033W*, show growth similar to the control (*RRP4 mtr4-E1033W*). Both Mtr4 R1030A and Mtr4 E1033W decrease helicase unwinding capability (Taylor *et al*. 2014). However, the Mtr4 R1030A variant is also implicated in disrupting target discrimination by the TRAMP complex, potentially by disrupting preferential polyadenylation by Trf4 (Taylor *et al*. 2014). Therefore, the negative genetic interaction observed for *rrp4-M68T rrp4-R1030A* cells further suggests that TRAMP function is impacted in *rrp4-M68T* cells. Taken together with our structural modeling data, we hypothesize that a stabilized interaction between the RNA exosome and Mtr4 is necessary for TRAMP association and the slightest perturbation, even a subtle destabilization at one contact point with the helicase, could disrupt this vital interaction between TRAMP and the complex. More biochemical studies could be performed to explore how changes within the EXOSC2-MTR4/Rrp4-Mtr4 interface impact the interaction with the TRAMP complex.

Our studies also show that *rrp4-M68T* mutant cells have distinct genetic interactions as compared to the *rrp4-G226D* cells. The *rrp4-G226D mtr4-R349E-N352E* double mutant cells surprisingly show improved growth at 37°C compared to either single mutant. Even more surprising is the synthetic lethality in cells expressing *rrp4-G226D* and either *mtr4* helicase mutant (*mtr4-R1030A* and *mtr4-E1033W*). These genetic interactions could suggest that the modeled SHRF amino acid substitution (Rrp4 G226D) has distinct in vivo consequences compared to the modeled multiple myeloma-associated substitution Rrp4 M68T. The Rrp4 G226D variant has decreased association with Mtr4 and the *rrp4-G226D* cells show transcriptomic differences from wild-type cells consistent with disrupted RNA exosome-Mtr4 interactions (Sterrett *et al*. 2021). Similarly, we observe decreased association between the Rrp4 M68T variant and Mtr4 and some RNA exosome target transcripts accumulate in *rrp4-M68T* cells that also accumulate in *rrp4-G226D* cells. However, notably, we do not detect any changes in select CUTs or 5.8S rRNA precursors in *rrp4-M68T* cells. We do, intriguingly, observe a significant decrease in the steady-state level of *INO1* mRNA in *rrp4-M68T* cells that is shared in the *rrp4-G226D* cells (Sterrett *et al*. 2021). Previous work characterizing the *rrp4-G226D* mutation suggested that the significant change in *INO1* mRNA levels could reflect defects in the cytoplasmic roles of the RNA exosome (Sterrett *et al*. 2021), though the exact molecular mechanism remains unknown. A comparison of the results obtained for *rrp4-M68T* and *rrp4-G226D* suggests that there may be some distinct defects in RNA exosome function in each of these mutants though they may also have some overlapping consequences in vivo, in part due to altered association with Mtr4. The difference in molecular consequences between the two *rrp4* variants could be attributed to the impact on RNA exosome complex integrity observed in *rrp4-G226D* cells, which was not observed in *rrp4-M68T* cells (Fig. 5b) (Sterrett *et al*. 2021).

The difference in severity of functional and molecular consequences that we observe for the *rrp4-M68T* and *rrp4-G226D* mutant models may partially explain the differences in disease pathology between SHRF patients with the mutation *EXOSC2 G198D* and the multiple myeloma patient with the mutation *EXOSC2 M40T*. The *EXOSC2 G198D* mutation was identified in SHRF patients through whole exome sequencing and classified as causing a novel Mendelian syndrome (Di Donato *et al*. 2016). In contrast, the *EXOCS2 M40T* mutation is a spontaneous, somatic mutation that likely cooccurred with a chromosome 9 duplication. Additionally, the patient with this *EXOSC2 M40T* mutation has several chromosomal aberrations that are a hallmark of multiple myeloma, suggesting that these *EXOSC2* mutations could be passenger mutations rather than a pathogenic driver of the multiple myeloma. Upon further analysis of the non-coding mutations in the patient harboring *EXOSC2 M40T*, we found a second mutation present in intron 1 of *EXOSC2* in this patient (Supplementary Fig. 6). This mutation *(EXOSC2 SNV chr9:130,693,915 T > G)* is predicted to alter the splice donor site and likely to result in a misprocessed mRNA or truncated protein. Through RNA-Seq data available in CoMMpass for this patient, we determined that the *EXOSC2 M40T* missense mutation and the splice donor mutation are expressed from the same allele. Interestingly, we calculate the allelic frequency of these two *EXOSC2* mutations to be very similar (0.2266 vs. 0.2191). This suggests that *EXOSC2* M40T and the *EXOSC2* splice donor mutation either cooccurred or that the splice donor mutation was selected for in response to the *EXOSC2 M40T* missense mutation, which could negatively affect cell growth and/or survival. As *EXOCS2* is an essential gene in 1,076/1,086 cancer cell lines in the Cancer Dependencies Map project (depmap.org) including all 19 myeloma cell lines in the dataset, a future approach would be to engineer the *EXOSC2* M40T mutation into myeloma cell lines to determine the effects on RNA exosome function as well as myeloma cell growth and survival.

As the *rrp4-M68T* cells show defects in RNA exosome function, likely through altered of interactions with the RNA helicase Mtr4 and the associated TRAMP complex, this EXOSC2 M40T substitution could be detrimental to the function of the human RNA exosome. Altering key cofactor interactions with the RNA exosome could impact the processing and degradation of target RNA transcripts such as small ncRNA species that have key regulatory roles in various cellular processes. Furthermore, the interaction between the RNA exosome and MTR4 has been suggested to resolve secondary DNA structures associated with strand asymmetric DNA mutagenesis that can lead to genome instability and chromosomal translocations particularly in plasma B cells (Lim *et al*. 2017). The high level of evolutionary conservation within the N-terminus of EXOSC2 that interacts with MTR4 (Fig. 1c and Supplementary Fig. 2) suggests that there could be evolutionary pressure to maintain the integrity of certain sequences within EXOSC2 that specifically interact with key cofactors. Taking a genetic approach to assess different *EXOSC* missense mutations associated with human diseases can help unravel different consequences in specific interactions of the essential RNA exosome complex.

Utilizing the yeast genetic model system, we have characterized an *EXOSC2* mutation found in a multiple myeloma patient. However, this mutation was one of several mutations identified in genes encoding structural subunits of the RNA exosome in the CoMMpass study (Supplementary Fig. 1). The frequency of multiple myeloma mutations identified in the *DIS3* catalytic exosome gene suggests that there is an important link between multiple myeloma and the RNA exosome. By modeling identified *EXOSC* mutations in the budding yeast system, we can examine whether these mutations impair the function of the essential RNA exosome and provide a deeper understanding of the role that this conserved complex may have in cancer pathologies. While it is unlikely that the identified *EXOSC* mutations drive the multiple myeloma disease, our study here clearly shows that these mutations have in vivo consequences for the conserved and essential RNA exosome-Mtr4 interaction. In addition, by studying other models of *EXOSC* disease-linked mutations, such as those identified in RNA exosomopathy patients, we can provide insight into the biological pathways that are altered in these different disorders. As more pathogenic mutations are uncovered in *EXOSC* genes via accelerated genomic screening of patients, generating in vivo models to explore the consequences of these changes can help to define the most critical interactions of the complex with various cofactors and thus expand our understanding of the biological functions of this essential RNA processing and degradation complex.

## Supplementary Material

jkad049_Supplementary_Data

## Data Availability

Strains and plasmids summarized in Supplementary Table 1 and Supplementary Table 2 are available upon request. Genomic data from CoMMpass are available at dbGaP with the accession number phs000748.v7.p4. The authors affirm that all data necessary for confirming the conclusions of the article are present within the article, figures, and tables. Supplemental material available at G3 online.
