# Supplementary material for: In vivo characterization of the critical interaction between the RNA exosome and the essential RNA helicase Mtr4 in Saccharomyces cerevisiae:
Source: G3 (Bethesda). 2023 Mar 2;13(8):jkad049. doi: 10.1093/g3journal/jkad049 (PMC10411580; doi:10.1093/g3journal/jkad049)

Supplementary Figure S1. RNA exosome subunit gene mutations identified in newly diagnosed multiple myeloma patients in the CoMMpass study. Cartoon depicting the human nuclear RNA exosome ribonuclease complex. The nine structural subunits, EXOSC1-9, and the catalytic exo/endoribonuclease, DIS3, are labeled. The nuclear RNA exosome has an additional 3'-5' exonuclease, EXOSC10 (Rrp6 in yeast), that associates with the complex and aids in nuclear RNA targeting and processing. The associated nuclear cofactors Mpp6 and Rrp47 are also depicted. The ongoing longitudinal Multiple Myeloma Research Foundation (MMRF) study "Relating Clinical Outcomes in Multiple Myeloma to Personal Assessment of Genetic Profile" (CoMMpass) [ClinicalTrials.gov Identifier NCT01454297] identified mutations in *EXOSC2, EXOSC8, EXOSC10* and *DIS3* within multiple myeloma patients upon diagnosis (the encoded subunits are colored pink in the cartoon depiction). Whole genome sequencing was performed on 940 newly diagnosed patients through the CoMMpass study. Within that population, rare single nucleotide variants (SNVs) were identified in the structural RNA exosome cap and core genes, *EXOSC2* and *EXOSC8.* Additionally, SNVs were identified in *EXOSC10*. Mutations in *DIS3* were identified more commonly within newly diagnosed patients in CoMMpass. The number of newly diagnosed patients with SNVs or mutations in *EXOSC2, EXOSC8, EXOSC10* or *DIS3* identified through CoMMpass is listed in the figure next to the corresponding subunit.

Supplementary Figure S2. ConSurf analysis of EXOSC2 and Rrp4 reveals conservation at interface with MTR4/Mtr4. The ConSurf server tool (Ashkenazy *et al.* 2010; Celniker *et al.* 2013; Ashkenazy *et al.* 2016) was used to assess the conservation of both EXOSC2 (A) and Rrp4 (B). Residues are colored based on calculated conservation scores representing a relative measure of evolutionary conservation. Conservation scores range from 1 (blue) to 9 (pink). Calculated conservation scores that do not pass statistical tests are marked as having insufficient data and are colored yellow in the structure. Conservation score colors are mapped onto structures of EXOSC2 in the mammalian RNA exosome (PDB 6D6R) and Rrp4 in the budding yeast RNA exosome (PDB 6FSZ). Both structures include the RNA helicase MTR4/Mtr4 (purple). Zoomed insets show the conservation of the region of EXOSC2 that includes Met40 and the corresponding Rrp4 Met68.

Supplementary Figure S3. The steady-state level of mature and precursor 5.8S rRNA in *rrp4-M68T* cells is similar to wild-type, control cells. Total RNA from mutant cells (*rrp4-M68T*, *rrp4-G226D*, *mtr4-1)* and the corresponding wild-type control cells (*RRP4* and *MTR4*) grown at 37°C was extracted analyzed by RT-qPCR with primers that amplifies the mature 5.8S rRNA (AC9791/9792; red) or with primers that flank the 5.8S-ITS2 junction (AC9793/9794; orange) to detect 7S pre-rRNA. The 7S pre-rRNA is normally processed to mature 5.8S rRNA by 3'-5' decay of the internal transcribed spacer 2 (ITS2) via the nuclear RNA exosome (Mitchell *et al.* 1996; Allmang *et al.* 1999). The simplified schematics to the right illustrate the processing steps of 7S rRNA precursor following endonucleolytic cleavage from the larger 27S precursor (indicated by white triangles). The locations of the primer sets are denoted on the simplified schematic. The steady-state level of mature 5.8S and pre-5.8S rRNA is not significantly increased in *rrp4-M68T* cells (denoted in pink) compared to control *RRP4* cells. The steady-state levels of these rRNA species are significantly increased in *rrp4-G226D* cells (denoted in gray) as previously reported (Sterrett *et al.* 2021). Furthermore, the steady-state level of pre-5.8S rRNA is significantly increased in the *mtr4-1* cells when compared to control *MTR4* cells while the level of mature 5.8S rRNA is not significantly different between the *mtr4-1* and *MTR4* cells (denoted in purple). RNA isolation and RT-qPCR were performed as described in *Materials and Methods.* Statistical significance of the RNA levels in *rrp4* variant cells relative to *RRP4* cells and in the *mtr4*-1 cells relative to *MTR4* cells is denoted by an asterisk (**p*-value $\leq$ 0.05; ***p*-value $\leq$ 0.01, ****p*-value $\leq$ 0.001).

Supplementary Figure S4. Synthetical lethality of either *rrp4-M68T mtr4-R349E-N352E* and *rrp4-M68T mtr4-R1030A* double mutant cells is rescued by wild-type plasmid. The *rrp4*Δ *mtr4*Δ cells containing *RRP4 URA3* and *MTR4 URA3* maintenance plasmids were transformed with empty vectors, or the *MTR4/mtr4 HIS3* plasmids and the *RRP4/rrp4 LEU* plasmids. Cells were grown overnight and serially diluted and spotted onto Ura^- Leu^- His^-minimal media plates, which select for cells that contain *URA3* maintenance plasmids, the *RRP4*/*rrp4 LEU2* plasmid, and the *MTR4/mtr4 HIS3* plasmid. Cells were also spotted onto 5-FOA Leu^- minimal media plates, which selects for cells that lack the *URA3* maintenance plasmids and contain only the *RRP4*/*rrp4 LEU2* and *MTR4/mtr4 HIS3* plasmids. The plates were incubated at 30°C for 3 days.

Supplementary Figure S5. Extended liquid growth curve of *rrp4-M68T mpp6Δ* and *rrp4-M68T rrp47Δ* cells. The *rrp4*Δ *mpp6*Δ or *rrp4*Δ *rrp47*Δ cells expressing *RRP4* or *rrp4-M68T* were grown in liquid media at 37°C with optical density measurement used to assess cell density over time. Data shown are collected from four independent samples (n = 4). These growth curves are the source of the data displayed in Figure 6D carried out for a longer time course and used to quantify the doubling time presented in Figure 6E.

Supplementary Figure S6. Chr9- NC_000009.12 (130,693,760…130,704,894) schematic with multiple myeloma patient *EXOSC2* mutations. Gene schematic depicting the *EXOSC2* locus. The gene contains nine exons labeled in roman numerals. Coding sequence and the 5' and 3' UTRs are color coded in pink and light pink, respectively. The multiple myeloma patient mutation location is depicted in red. The missense mutation *chr9:130,693,910 c.119 T>C* is in exon I and results in the EXOSC2 Met40Thr substitution. The single nucleotide variant (SNV) *chr9:130,693,915 T>G* is a splice donor mutation within the first intron. This splice donor SNV is reported in NIH dsSNP (rs1430887213).

Strain/Plasmid	Description	Source
rrp4Δ (yAV1103)	*MATα; ura3Δ0; leu2Δ0; his3Δ1; lys2Δ0; RRP4::neoMX (G418+); [RRP4; URA3]*	Losh 2018(Losh 2018)
rrp4Δmpp6Δ (ACY2471)	*MATα; ura3Δ0; leu2Δ0; his3Δ1; RRP4::neoMX (G418+); [RRP4; URA3]; MPP6::natMX4*	Sterrett Enyenihi et al. 2021
rrp4Δrrp47Δ (ACY2474)	*MATα; ura3Δ0; leu2Δ0; his3Δ1; RRP4::neoMX (G418+); [RRP4; URA3]; LRP1::natMX4*	Sterrett Enyenihi et al. 2021
RRP45-TAP (ACY2789)	*MATa; ura3Δ0; leu2Δ0; his3Δ1; met15Δ0; RRP45-TAP:HIS3MX6*	Ghaemmaghami et al. 2009
rrp4Δmtr4Δ (ACY2536)	*MATα; ura3-; leu2-; his3-; trp1-; LYS+; GAL+; ADE+; MTR4::natMX4; [pAC3714; MTR4; RRP4; URA3; CEN]; RRP4::neoMX*	Sterrett Enyenihi et al. 2021
mtr4Δ (ACY2532)	*MATα; ura3-; leu2-; his3-; trp1-; LYS+; GAL+; ADE+; MTR4::natMX4; [pAC3714; MTR4; RRP4; URA3; CEN]*	This Study
pRS315 (pAC3)	*CEN6, LEU2, ampR*	Sikorski and Hieter 1989
pRS313 (pAC1)	*CEN6, HIS3, amp^R*	Sikorski and Hieter 1989
pAC3656	*RRP4-Native 3' UTR* in pRS315, *CEN6, LEU2, ampR*	Sterrett Enyenihi et al. 2021
pAC3669	*RRP4-2xMyc-Native 3' UTR* in pRS315, *CEN6, LEU2, ampR*	Sterrett Enyenihi et al. 2021
pAC3659	*rrp4-G226D-Native 3' UTR* in pRS315, *CEN6, LEU2, ampR*	Sterrett Enyenihi et al. 2021
pAC3672	*rrp4-G226D-2xMyc-Native 3' UTR* in pRS315, *CEN6, LEU2, ampR*	Sterrett Enyenihi et al. 2021
pAC3714	*MTR4, RRP4, CEN6, URA3, amp^R*	Sterrett Enyenihi et al. 2021
pAC3719	*MTR4-2xFLAG, CEN6, HIS, amp^R*	Sterrett Enyenihi et al. 2021
pAC4096	*MTR4-Native 3'UTR* in pRS313, *CEN6, HIS3, ampR*	Sterrett Enyenihi et al. 2021
pAC4099	*mtr4-F7A-F10A-Native 3'UTR* in pRS313, *CEN6, HIS3, ampR*	Sterrett Enyenihi et al. 2021
pAC4103	*mtr4-1-(mtr4-C942Y)-Native 3'UTR* in pRS313, *CEN6, HIS3, ampR*	This Study
pAC4104	*mtr4-R1030A-Native 3'UTR* in pRS313, *CEN6, HIS3, ampR*	This Study
pAC4105	*mtr4-E1033W-Native 3'UTR* in pRS313, *CEN6, HIS3, ampR*	This Study
pAC4206	*rrp4-M68T-Native 3' UTR* in pRS315, *CEN6, LEU2, ampR*	This Study
pAC4207	*rrp4-M68T-2xMyc-Native 3' UTR* in pRS315, *CEN6, LEU2, ampR*	This Study

Description	Sequence (5'-3')	Name
INO1 mRNA Fwd	TTGGACTGCAAATACTGAGAGG	AC9303
INO1 mRNA Rev	AAGATCGTGGAAGGAGCAATC	AC9302
CUT501 ncRNA Fwd	GCTAGCACCTGTTGCTGTAAT	AC9255
CUT501 ncRNA Rev	GGTTCAACGTTGCAGGATCT	AC9254
CUT770 ncRNA Fwd	AAACAACCCGCTAGTGTGAC	AC9262
CUT770 ncRNA Rev	AGAGCAACTCACTGCAAAGG	AC9263
CUT896 ncRNA Fwd	CCCAGAGGCAAAGATGTTAAGT	AC9257
CUT896 ncRNA Rev	ATCAGCAGGTGTCATGTTACAG	AC9256
pre-TLC1 ncRNA Fwd	CCGCCTATCCTCGTCATGAAC	AC7594
pre-TLC1 ncRNA Rev	GTATTGTAGAAATCGCGCGTAC	AC7593
mature *TLC1 ncRNA* Fwd	AAGGCAAGGGTGTCCTTTCT	AC6420
mature *TLC1 ncRNA* Rev	TTCCGCTTGGAAAATAATGC	AC6421
3' extended *U4 snRNA* Fwd	ATCCTTATGCACGGGAAATACG	AC5722
3' extended *U4 snRNA* Rev	AAAGAATGAATATCGGTAATG	AC5723
3' extended *snR33 snoRNA* Fwd	AAGCGACCTTTCTTCGCA	AC9787
3' extended *snR33 snoRNA* Rev	TTCGCTTCTGGTTACTGCAA	AC9788
5.8s rRNA mature Fwd	CAACAACGGATCTCTTGGTTCT	AC9791
5.8s rRNA mature Rev	GAAATGACGCTCAAACAGGCA	AC9792
5.8s-ITS2 rRNA marginal Fwd	CGAATCTTTGAACGCACATTGC	AC9793
5.8s rRNA precursor 3' Rev	GGAAATGACGCTCAAACAGG	AC9794
ALG9 mRNA Fwd	CACGGATAGTGGCTTTGGTGAACAATTAC	AC5067
ALG9 mRNA Rev	TATGATTATCTGGCAGCAGGAAAGAACTTGGG	AC5068
PGK1 mRNA Fwd	CTGCTTTGCCAACCATCAAGT	AC2307
PGK1 mRNA Rev	GCAACTGGAGCCAAAGAGTATTTT	AC2308

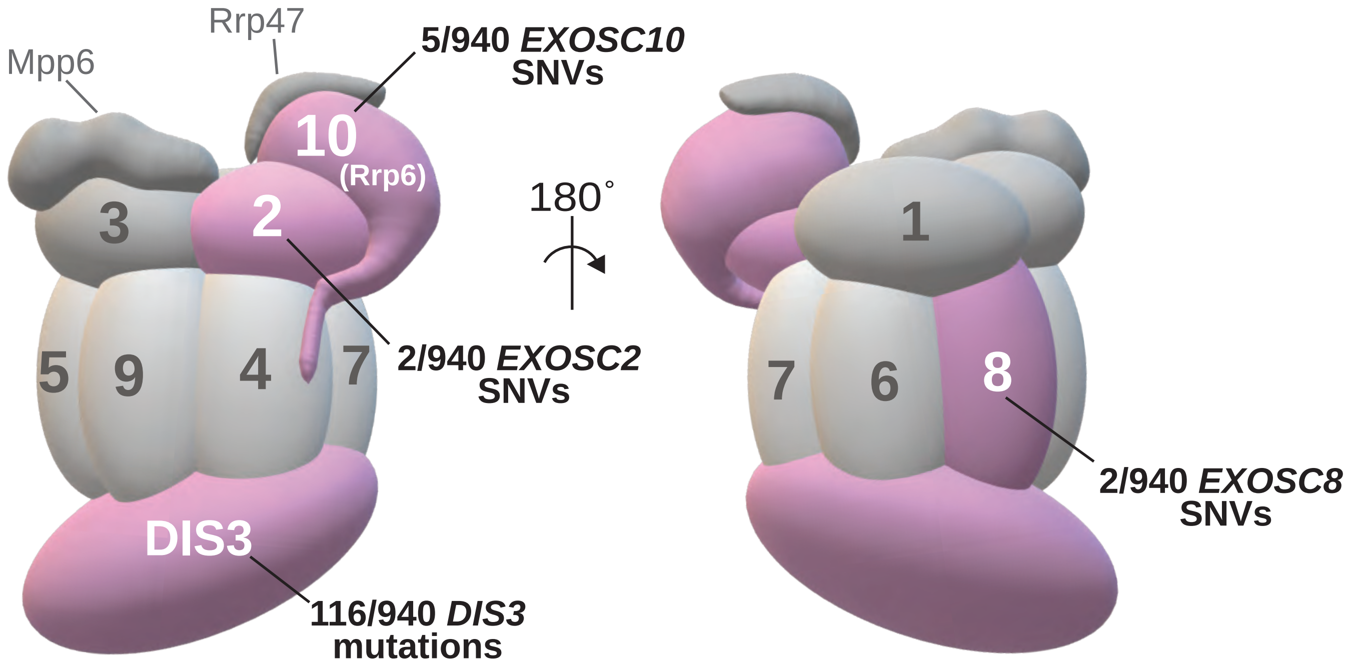
Rrp47
Mpp6
5/940 *EXOSC10* SNVs
10
(Rrp6)
3
2
180˚
1
5
9
4
7
2/940 *EXOSC2* SNVs
7
6
8
2/940 *EXOSC8* SNVs
DIS3
116/940 *DIS3* mutations

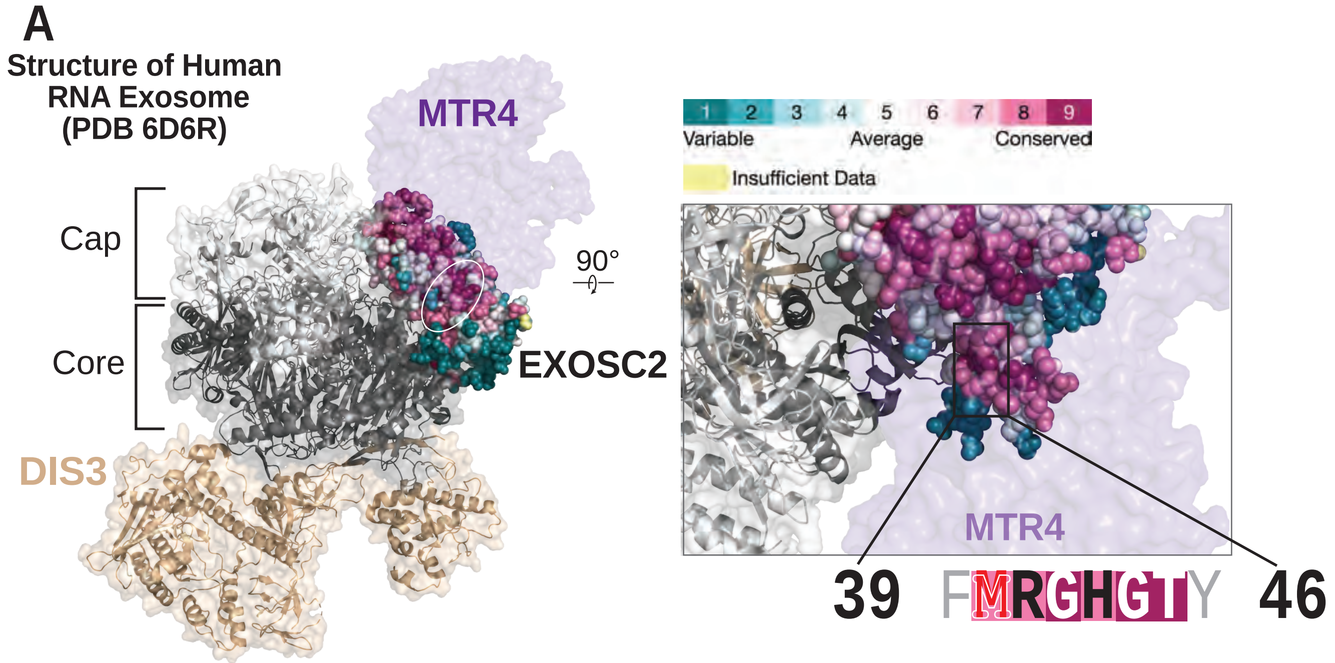

A
Structure of Human
RNA Exosome
(PDB 6D6R)
MTR4
Cap
Core
DIS3
EXOSC2
90°
1 2 3 4 5 6 7 8 9
Variable
Average
Conserved
Insufficient Data
MTR4
39
FMRGHGTY
46

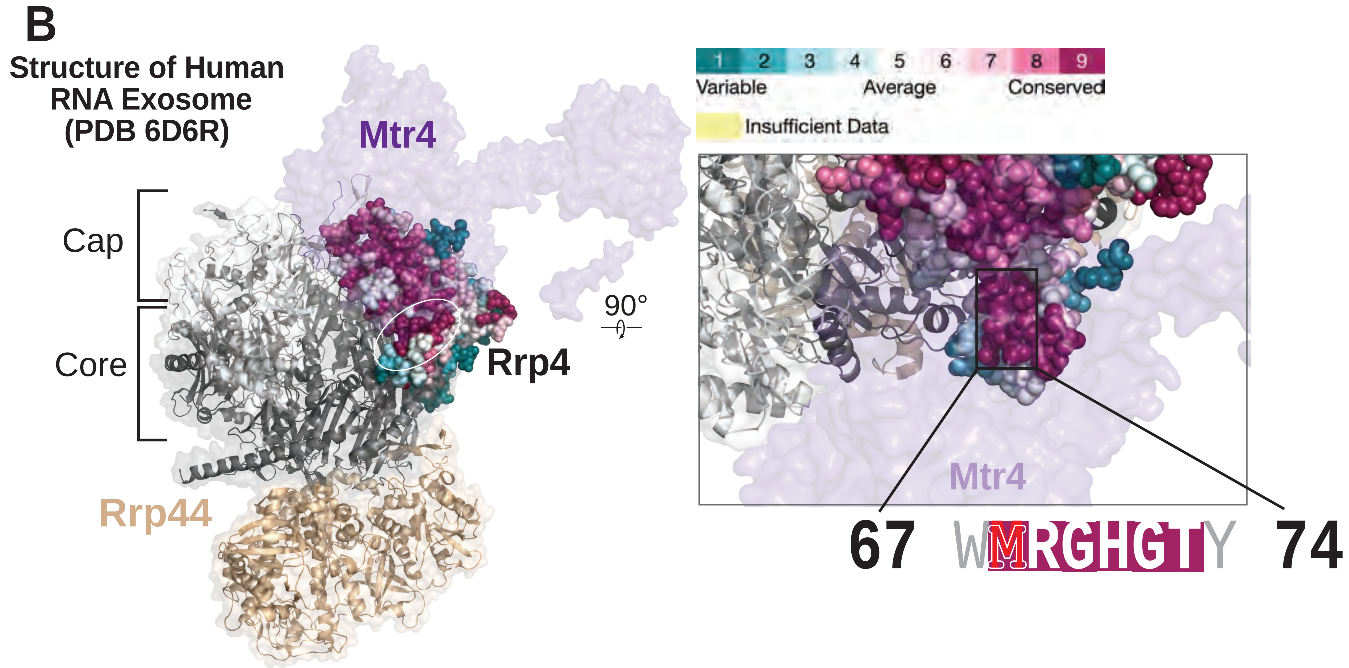

B
Structure of Human
RNA Exosome
(PDB 6D6R)
Mtr4
Cap
Core
Rrp44
Rrp4
90°
1 2 3 4 5 6 7 8 9
Variable
Average
Conserved
Insufficient Data
Mtr4
67
WMRGHGTY
74

Figure S3

Sterrett *et al.*

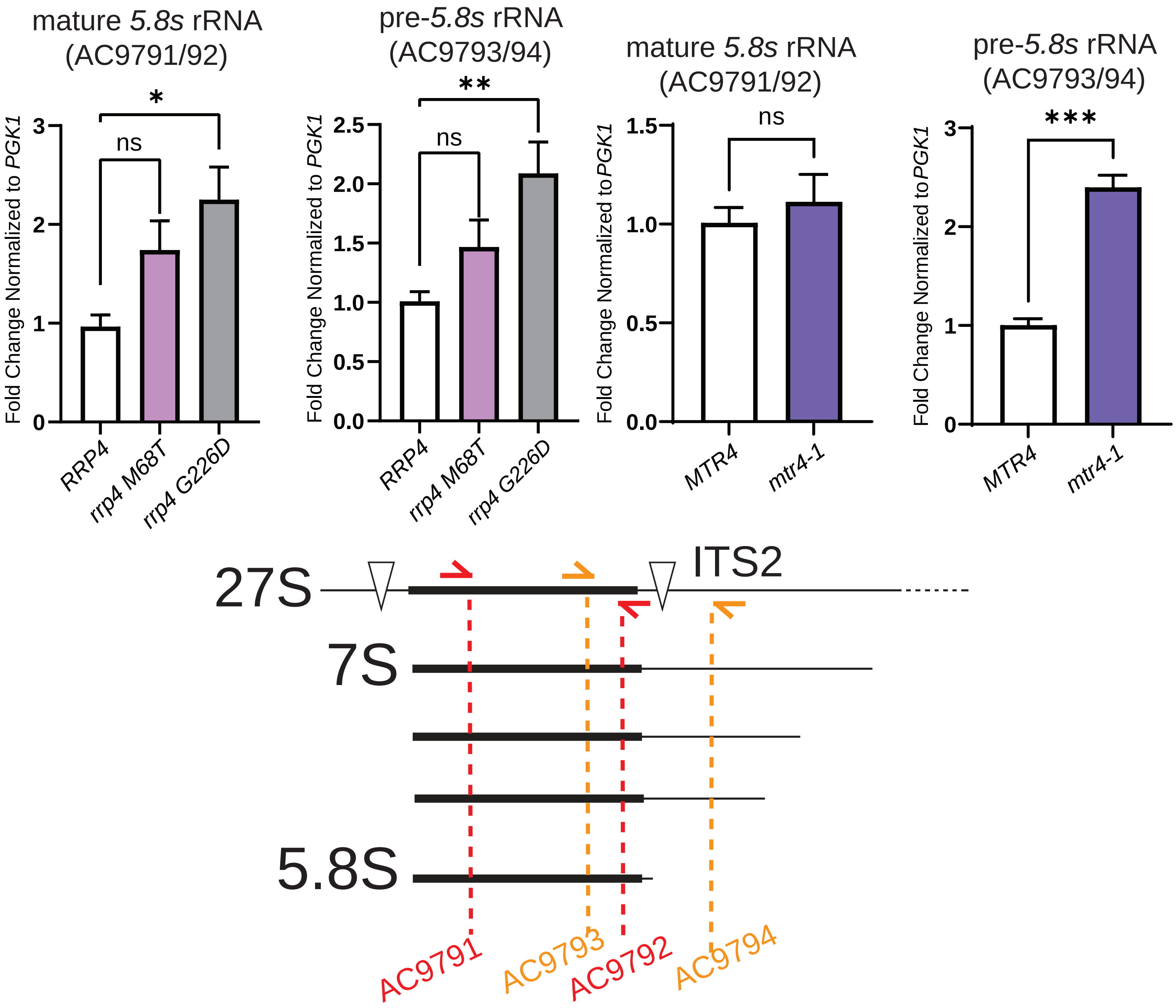

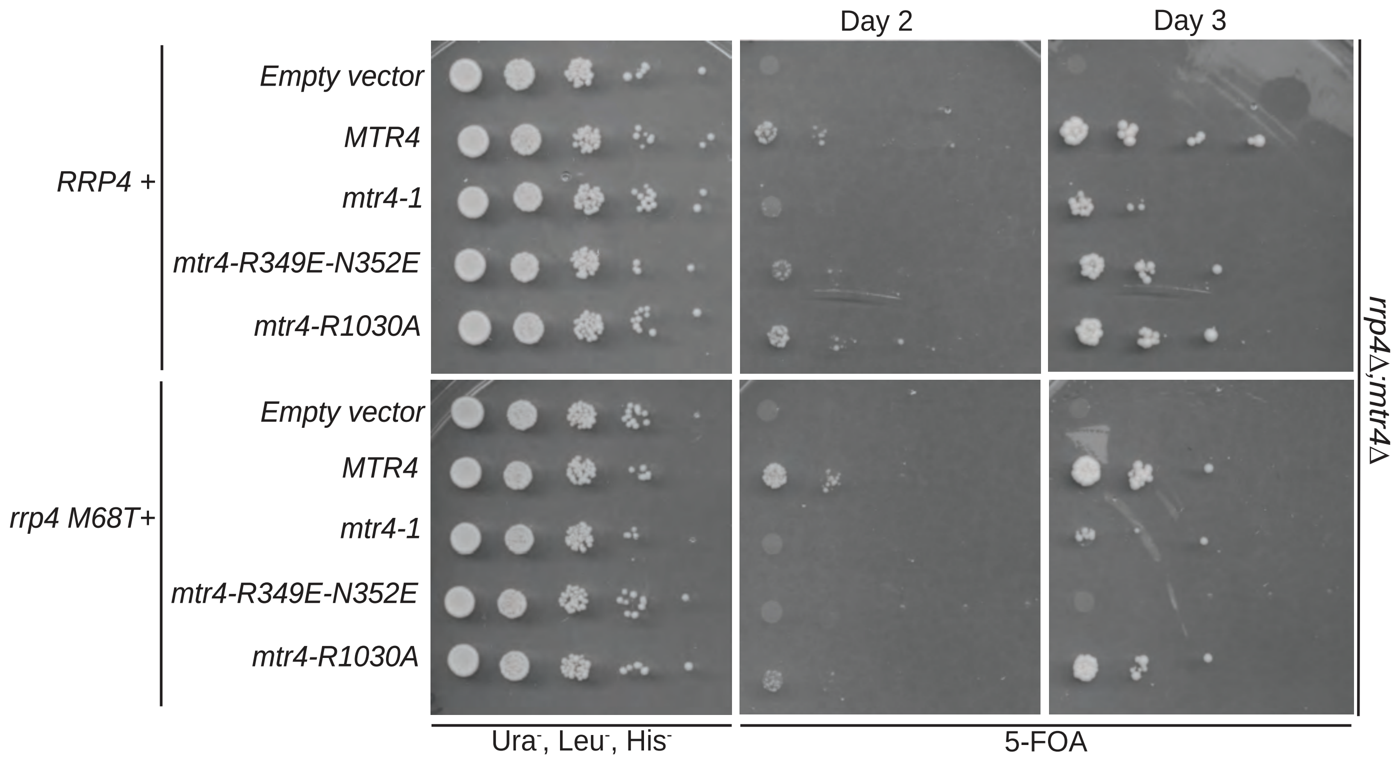
Day 2
Day 3
RRP4 +
Empty vector
MTR4
mtr4-1
mtr4-R349E-N352E
mtr4-R1030A
rrp4 M68T+
Empty vector
MTR4
mtr4-1
mtr4-R349E-N352E
mtr4-R1030A
rrp4Δ;mtr4Δ
Ura-, Leu-, His-
5-FOA

Figure S5

Sterrett *et al.*

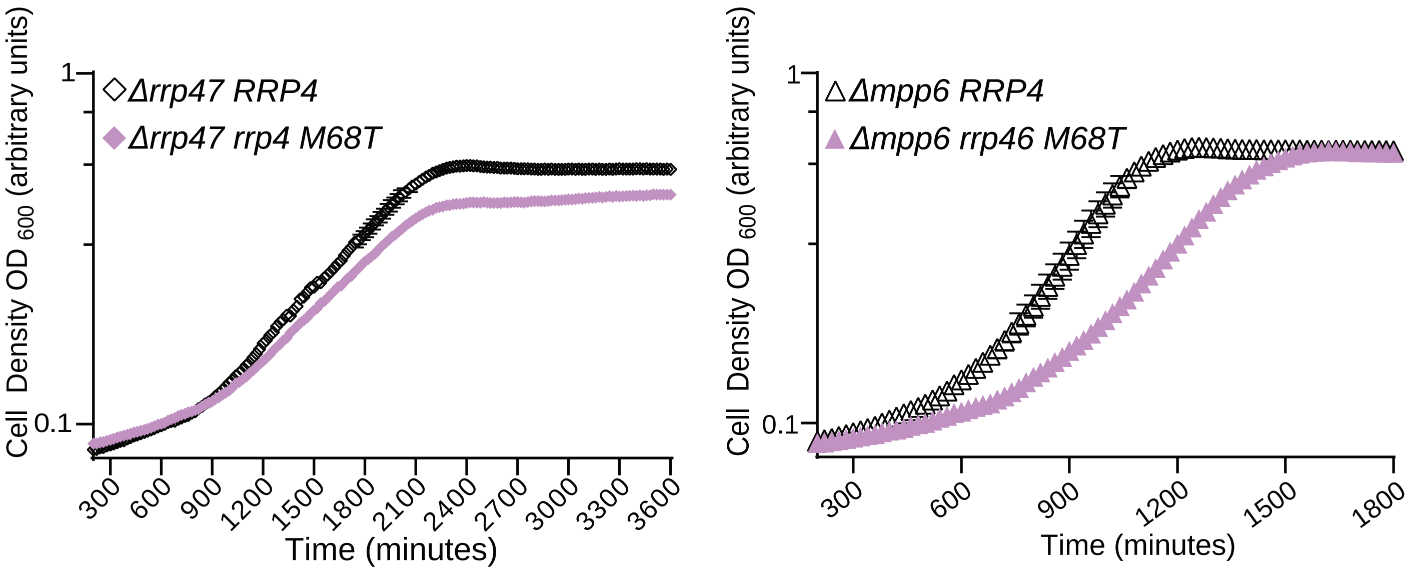

Figure S6

Sterrett *et al.*

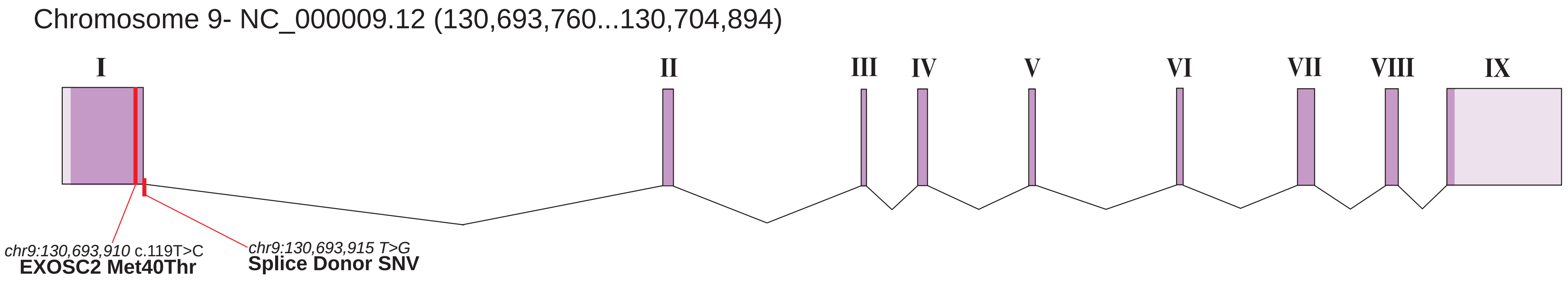

Supplement: jkad049_Supplementary_Data [file jkad049_supplementary_data.pdf]
